# Patient Participation During Nursing Bedside Handover: A State-of-the-Art Review

**DOI:** 10.3390/nursrep15120438

**Published:** 2025-12-10

**Authors:** Paulo Cruchinho, Gisela Teixeira, Pedro Lucas, Filomena Gaspar, María Dolores López-Franco

**Affiliations:** 1 Inter University Doctoral Studies in Comprehensive Care and Health Services (UJA-UDL-UVIC), University of Jaén, 23071 Jaén, Spain; 2 Nursing Research Innovation and Development Centre of Lisbon (CIDNUR), School of Nursing, University of Lisbon, 1600-190 Lisbon, Portugal; gteixeira@esel.pt (G.T.); prlucas@esel.pt (P.L.); mfgaspar@esel.pt (F.G.); 3 Nursing Administration Department, School of Nursing, University of Lisbon, 1600-190 Lisbon, Portugal; 4 CTS-464 Nursing and Innovation in Healthcare, Department of Nursing, University of Jaén, 23071 Jaén, Spain; mlfranco@ujaen.es

**Keywords:** nursing handover, patient participation, patient empowerment, patient engagement, patient involvement, patient-centered nursing, comprehensive health care, review, nursing administration research

## Abstract

**Background:** Patient participation during Nursing Bedside Handover (NBH) is a dyadic interaction between the patient and nurses that allows the patient to participate, either passively or actively, in communication activities and nursing care. **Objective:** This state-of-the-art (SotA) review aimed to synthesize current knowledge on patient participation during NBH and identify future directions for bedside handover research. **Methods:** The literature search was conducted through PubMed, CINAHL Complete, and Scopus, and was supplemented by citation searching. Search was limited to peer-reviewed scientific articles using any empirical study design that addressed patient participation during NBH published in English by August 2025. The quality of the included studies was assessed using the Mixed Methods Appraisal Tool. **Results:** A total of 50 primary research articles were included and examined using the method of constant comparisons. The synthesized data were categorized into three main themes: (a) Domain of distinctive nature and attributes of patient participation during NBH; (b) domain of nurses’ practices and influencing factors of patient participation during NBH; and (c) domain of strategies and impacts of increasing patient participation during NBH. Within each domain, research trends were identified concerning patient participation in NBH. Future research directions are presented within each domain. **Conclusions:** The findings of this review may provide new insights into developing complex interventions aimed at increasing patient participation in NBH by nurses, namely with the use of co-design strategies, as well as the adoption of transfer protocols that incorporate informational and interactional components and assessment tools to measure patient participation in NBH.

## 1. Introduction

Increasingly, international healthcare policy has emphasized the importance of patient and family participation in improving the quality and safety of care as the only constants in increasingly complex health systems. This is due to the repertoire of critical information from their care experiences, which cannot be replaced or replicated by health professionals, managers, or researchers, but can be shared when they are involved and empowered by professionals. An example is the World Health Organization Program, Patients for Patient Safety (PFPS) [1], under the A Decade of Patient Safety 2021–2030 initiative, which aims to accomplish the following: (1) have patients take responsibility for their care; (2) bring the voice of patients to the forefront of healthcare; and (3) facilitate partnerships between patients, families, the community, and health professionals. Another example is a strategic objective of the Global Patient Safety Action Plan 2021–2030 [2], titled, Engage and Empower Patients and Families to Help and Support the Journey to Safer Health Care, which enables patients, families, and caregivers to act as vigilant observers who can alert professionals when new needs arise. One of the actions under this strategic objective involves structuring healthcare service processes to support information sharing, care planning, shared decision-making, and implementing patient-centered tools [2,3,4].

## 2. Background

Since the publication of Communication During Patient Hand-Overs [5], in which the World Health Organization (WHO) recommended patient and family participation as a strategy to increase handover safety, several studies and experiences have been published worldwide over the past two decades concerning the transition from office-based or station-based shift handover to the Nursing Bedside Handover (NBH). Despite the lack of a conceptual definition for NBH [6,7], this type of handover is characterized by its patient-centered [8,9] and family-centered approach [10,11] due to the direct involvement of patients [12,13], families [14,15,16], the encouragement of nurses to ask questions [17,18], and for patients to express their opinions or make comments during handover [19]. It is also characterized by information sharing with patients [20,21] and families [11,22], which may require nurses to view the patient as a source of information [23], and by discussion [24,25] and joint care planning with the patient and family for the next shift [26,27,28]. It also includes monitoring the patient’s condition [29] and carrying out safety checks of their clinical devices [30,31]. It is, therefore, a type of critical communication for patient safety, during which information, responsibility, and accountability for patient care are transferred between nurses [32], and it seeks patient contribution during this transfer, strengthening the safety culture of healthcare organizations [33].

Several outcomes have been associated with this handover practice. Regarding patient outcomes, evidence shows benefits in terms of patient satisfaction [34,35], reduced patient anxiety due to a greater understanding of care [35,36], enhanced interactive communication between patients and nurses [37], increased active involvement of patients in care planning and greater autonomy in this process [34,35,36,38], the development of a stronger partnership between patients and nurses [37], improved perceptions of safety in nursing care [35,39], more effective information/knowledge sharing [34], reduced risk of information omission [36], better preparation for hospital discharge [38], and overall improvements in the quality of nursing care [35]. Positive outcomes have also been reported for nurses and organizations. For nurses, these include stronger perceptions of patient safety, increased patient-involving behaviors, increased information sharing between nurses and patients [34], and improved professionalism in nurses [37]. At the organizational level, NBH has been linked to greater humanization of care [34], optimization of nursing care organization [37], and improved hospital efficiency through increased staff compliance, reduced overtime, lower costs, decreased call-light usage, fewer nursing care tasks, and improved completion of documentation [35].

Although NBH enables nurses to triangulate verbally shared information with visual assessment of the patient—while also involving the patient in handover communication and facilitating earlier prioritization of needs during the shift [40]—several disadvantages have been identified. These include a decreased sense of collegiality among nurses, since they now only hand over patients assigned to them and may not know the other patients on the ward [37,41]; the risk of disturbing patients’ sleep [36]; and the possibility of breaching confidentiality. However, this last concern is often less significant for patients, particularly when they are included in decision-making and feel recognized as partners in care [37]. In addition, evidence remains inconsistent regarding the duration of handovers [35,37], length of stay, the rate of unplanned readmissions, and adverse nursing-sensitive outcomes such as patient falls, pressure ulcers, and medication errors [34]. Successful implementation of NBH seems to depend largely on nurses’ training, the prevailing nursing culture, and available resources [35].

Consequently, many of the positive outcomes of patient and family involvement in NBH may be undermined by barriers such as time constraints [34], greater concern among nurses than patients regarding confidentiality [35,38,41,42], perceptions of increased workload [34], declining nurses’ compliance over time [34], cognitive load [34], and variability in implementation practices [34,35,38]. McClosket et al. [38], in a systematic review synthesizing the best available evidence on patients’, family members’, and nurses’ experiences with NBH, concluded that such barriers can limit patients’ active participation during NBH—asking or answering questions, correcting inaccuracies, or listening to shared information—and thus compromising safety. The role of patients and family members during NBH is to contribute relevant clinical information related to care or its evolution, which can influence patient safety [23]. It is acknowledged that patients who are asleep, confused, comatose, in isolation, unable to communicate in English, or affected by other conditions cannot play an active role in the NBH [43]. However, patients who do participate report greater awareness of their connection with nurses, which supports their involvement in their own care [37]. Importantly, the way nurses approach patients during NBH and the degree to which their practice is patient-centered remain potential barriers [23].

Neglecting and excluding patients from handover communication has been identified as a major barrier to NBH implementation [41]. Patient participation in NBH requires nurses to involve them in handover communication [44] and depends on the nurses’ efforts to integrate patients into the handover process [38]. This practice provides a set of intentional opportunities to involve the patient in assessing signs and symptoms, choosing or deciding on care activities, sharing information, and setting goals [45]. However, Forde et al. [40], in an integrative literature review, concluded that NBH does not automatically lead to patient participation in the handover. Instead, most handover content still reflects a biomedical model, delivered retrospectively rather than prospectively, and focuses little on patients’ priority needs or the ongoing care plan, thereby limiting inclusion [40]. Similarly, Tobiano et al. [23], in a systematic review on patient participation in shift-to-shift information transfer, distinguished between two NBH categories: (1) nurse-centered and (2) patient-centered handover. To improve patient safety, interventions aimed at enhancing handover should address barriers both before and after implementation [37].

Nursing handover communication is a key element for ensuring patient safety and continuity of nursing care [46], providing a valuable opportunity for nurse managers to enhance the centrality of nursing care based on patient involvement in the NBH [35,37]. To address the degree of patient participation, it is recommended to implement nurse-centered interventions, supported by a complex intervention research framework that accounts for the multiple needs of patients, nurses, and clinical contexts [47]. Such interventions should include safety-related content [37], adaptive strategies tailored to different needs, and links between clinical outcomes, process outcomes, and implemented nursing systems [47]. Regarding clinical outcomes, it is recommended that NBH implementation measure its impact on nursing-sensitive outcomes [35,48]. Furthermore, interventions should focus on strengthening nurses’ communication skills [19], not only to align with patient preferences and expectations for participation [19,23], but also to prevent confidentiality breaches during handover [19]. Given the complexity of NBH, it is also recommended that these interventions should be designed and conducted based on theories of organizational change [39].

The design and implementation of NBH thus provides an opportunity for nurses to highlight recent professional advancements and promote patient participation aligned with their preferences [19]. Although several reviews have examined the effectiveness of NBH implementation, none have comprehensively systematized knowledge about patient participation from a theoretical and conceptual standpoint, aimed at identifying models, mechanisms, and foundations for developing interventions to improve NBH. Because enhancing patient-centered care and reducing information gaps are critical to ensuring safety, nursing management must develop a thorough understanding of this phenomenon [49].

### 2.1. Theoretical Framework

This state-of-the-art (SotA) review is based on the Model for Developing Complex Interventions in Nursing by Corry et al. [50]. According to this model, the “synthesis of existing empirical evidence” (p. 2382), together with the scope of nursing practice, is an essential element in developing any complex nursing intervention, particularly for identifying the target problem, the general objective of the intervention, the key guiding principles, and for defining the intervention model. We define a complex nursing intervention as the deliberate attempt to introduce new patterns of collective nurse action or modify existing ones [51]. Thus, this review sought to synthesize evidence on patient participation during nursing handover communication, aiming to inform the development of interventions to improve NBH and guide future research.

### 2.2. Rationale and Aim of the Study

Based on Corry et al.’s model [50], this review aims to synthesize what is known in the existing empirical literature on patient participation during NBH and identify future directions for bedside handover research. Consequently, this theoretical framework provided an organized lens through which researchers examined current knowledge on patient participation in nurse handover communication, aiming to support nurse managers in the implementation and maintenance of this practice. A SotA review was chosen because it helps justify future research directions. This review is important because it extends previous reviews focused on patient participation in NBH [52], providing evidence that can guide the design not only of organizational interventions for implementing NBH, but also of future research in the field of inquiry.

## 3. Materials and Methods

The SotA review is a type of literature review focused on the most current issues of a specific research topic and is, therefore, limited to the latest developments [53], which is fundamental to support hypotheses and objectives of future research studies [54], in projects, theses, dissertations, and other future research activities [55]. In the nursing field, it is defined as a type of review that addresses only current and contemporary topics highlighted by the recent scientific literature [56]. It is a type of review that informs, “this is where we are now in our current understanding of this topic. This is how we got here. This is where we could go next” [57], and helps set priorities and highlight areas for future research [56]. This review is part of a broader project on the effectiveness of a complex intervention for improving patient safety by promoting patient participation in nursing handover. It followed the description of Grant and Booth [58] for SotA reviews, characterized by its focus on current issues and the identification of new perspectives for future research, and the six-step process proposed by Barry et al. [59], which includes the following: (1) determining the initial research question and field of study; (2) establishing the review period; (3) finalizing the research question(s) to reflect the defined time period; (4) developing a search strategy and looking for relevant studies; (5) analyzing the found articles; and (6) providing a reflective description that explains how the researchers’ experience informed the interpretation of the review data. The study was conducted from January to September 2025. Since there is no known checklist for reporting a SotA review, and since this type of review is recognized as a form of narrative synthesis of knowledge [59], the Scale for Assessment of Narrative Review Articles (SANRA) was used to prepare this study (See Table S1). The following subsections present each of the steps of the SotA review.

### 3.1. Determination of the Initial Research Question and Field of Inquiry (Stage 1)

An initial literature search identified several studies that analyzed aspects of patient participation in NBH. However, a specific focus on the development of complex interventions was lacking. In addition, the existing information had not yet been reviewed and consolidated comprehensively. Therefore, the initial research question guiding this state-of-the-art review was, “What is known from the existing empirical evidence about patient participation during nursing handover communication that is relevant for developing interventions to improve NBH and guide future research?” Based on the model by Corry et al. [50], we defined “existing empirical evidence” as research data or information derived from observation, experimentation, or experience, published in peer-reviewed academic journals, which needs to be synthesized to identify key theories or principles that will guide the development of a complex intervention amenable to the nursing scope. The field of inquiry of this review is, therefore, patient participation in handover communication.

### 3.2. Determination of the Timeframe (Stage 2)

We identified the publication of Communication During Patient Hand-Overs [5] by the WHO in 2007 as a historical marker signaling the beginning of contemporary developments in this field [5]. However, research on NBH was characterized by terminological inconsistency in defining bedside communication during nursing handover [60]. Numerous terms have been used interchangeably, including the following: “bedside shift handover,” “bedside transfer,” “bedside clinical handover,” “clinical handover at the bedside,” “shift handover at the bedside,” “shift transfer at the bedside,” “shift report at the bedside,” “shift change report at the bedside,” “shift transfer at the bedside,” “shift handover at the bedside,” “shift report at the bedside,” “bedside admission record,” “bedside discharge record,” “shift report,” “shift change,” “nursing handover,” and “nursing care handover”. To maximize the relevance and applicability of the review findings, no date restrictions were applied. All peer-reviewed scientific literature published in English up to August 2025 was searched. Due to the absence of theoretical contributions—such as frameworks and theories that provide the basis for the researcher’s belief system and for ways of thinking about the problem studied [61]—which still exists today in the field of inquiry, we considered this timeframe to be appropriate [62].

### 3.3. Finalization of the Research Question to Reflect the Timeframe (Stage 3)

The initial research question was maintained.

### 3.4. Development of the Search Strategy to Identify Relevant Articles (Stage 4)

In this literature review, we deliberately adopted a narrow search strategy to capture highly relevant empirical research into the development of complex interventions focused on patient participation in NBH, and we limited our review to all study designs peer-reviewed and published in academic journals. On the contrary, articles that did not address patient participation during nursing handover were excluded. Literature reviews were excluded to avoid data duplication and to ensure that the synthesis focused solely on original empirical evidence directly addressing the review question. Gray literature was also excluded (conference proceeding papers, monographs, dissertations, theses, practice guidelines, and others) due to the absence of peer review. Lastly, we excluded quality improvement projects, research protocols, secondary research studies, commentary articles, and editorials. PubMed, CINAHL Complete, and Scopus databases were used to search for relevant information. PubMed and CINAHL Complete are considered two essential databases for conducting nursing literature reviews [63]. We opted for PubMed over MEDLINE, as it is a broader database [64]. These three sources were supplemented by a citation search. This included reference checking and forward citation searching using Google Scholar. To answer the review question, we conceptualized patient participation as a dyadic interaction between patients and nurses that enables patients to engage passively or actively in handover communication activities [65]. Examples of patient participation in NBH include the following: (1) a passive participation in situations where family members provided information on behalf of patients unable to participate during handover; (2) one-way communication when nurses provided information and knowledge directly to patients with little interaction; and (3) a nurse–patient interaction where both engage in dialogue [66]. The review was conducted using specific keywords and related terms. To increase specificity, the database search was limited to “Title/Abstract”. Given the conceptual inconsistency surrounding NBH, a variety of terms were used that were previously found in the scientific literature. These terms were pre-tested in PubMed to ensure they retrieved NBH-related articles. We combined the MeSH descriptors “Patient participation,” “Patient Handoff,” and “Nursing” with these terms. Table A1 presents the search expressions used for each database (Appendix A.1).

**Figure 1:**
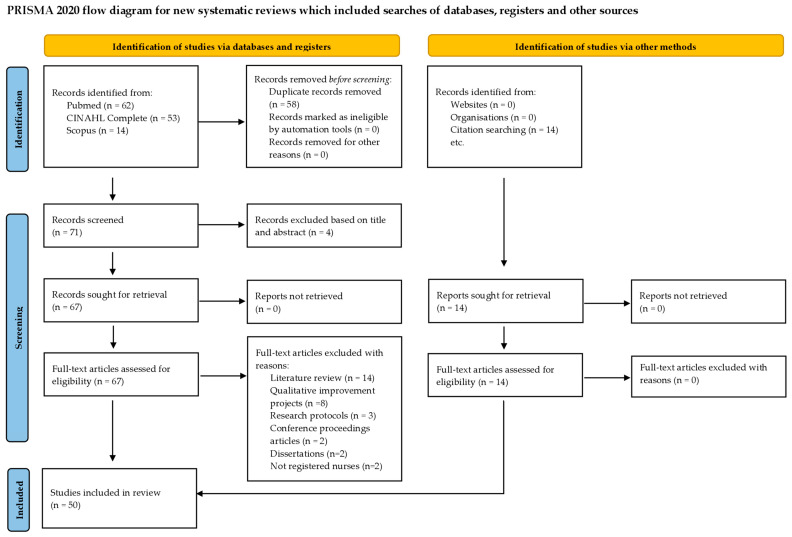
PRISMA flow diagram.

We obtained a total of 129 results from database searches, which were exported to the Rayyan platform [67] for screening and selection. After removing 58 duplicate studies, 71 articles remained for title and abstract screening. Four were excluded during this phase because they did not focus on patient participation in the NBH. Ultimately, 50 studies were selected, 14 of which were identified through a citation search. Screening and selection were performed by two independent reviewers (P.C. and G.T.), who first assessed titles and abstracts, followed by full-text screening of the sources to determine eligibility based on the inclusion and exclusion criteria described above. Disagreements between reviewers were resolved through discussion. The PRISMA flow diagram is shown in Figure 1. Although quality appraisal is not mandatory in SotA reviews, a quality appraisal of the studies included in this review was performed using the Mixed Methods Appraisal Tool (MMAT) [68] to minimize bias and to provide a good understanding of the evidence. Due to the diversity of study designs included in this review, the MMAT was considered the most appropriate evaluation tool, as it allows the assessment of five study categories: (1) Qualitative studies; (2) Quantitative randomized controlled trials; (3) Quantitative non-randomized studies; (4) Quantitative descriptive studies; and (5) Mixed methods studies [68].The MAAT is a valid [69] and reliable tool [70]. It includes five questions, depending on the study type, with three possible responses: “yes”, “can’t tell”, and “no”. For transparency and ease of comparison between studies, scores were assigned to the answers: “yes” = 2, “can’t tell” = 1, and “no” = 0. This scoring system allowed us to standardize the evaluation process, classifying studies into three levels of methodological quality: weak (score 0–3), moderate (4–7), and strong (8–10). Table A2 shows the results of the critical appraisal. We retained all studies because methodological quality scores were equal to or greater than 7 (Appendix A.2).

### 3.5. Analyses (Stage 5)

To ensure a rigorous SotA review, a systematic and interpretative approach was adopted. First, the text of the included articles was reviewed and mapped for relevant data to answer the review question. To compile the analysis corpus of this revision, data were manually extracted into a Word table: (1) authors and year of publication; (2) study aim(s); (3) study context; (4) study design and participants; and (5) results (see Table S2). To facilitate interpretation, an informative column was included on methodological quality based on Table A2.

All articles included in the analysis corpus were read thoroughly to achieve full familiarization with the literature. This familiarization process consisted of identifying similarities, ways of thinking that influenced perspectives on the field of inquiry, assumptions underlying changes in understanding, important decision points in the evolution of understanding, and gaps and assumptions in current knowledge. For example, key factors such as the presence of family members in the NBH, using handover protocols to standardize patient participation in the NBH, implementing educational interventions aimed at nurses, recognizing the importance of organizational handover policies, and valuing the active involvement of nurse managers were consistently highlighted throughout several studies. We also examined assumptions underlying nurses’ engagement behaviors, including that fears of breaching confidentiality and concerns about increasing the duration of the handover can be mitigated by implementing handover protocols aimed at patient engagement, and by learning communication strategies to manage sensitive information. We then traced the historical evolution of patient participation in NBH, identifying pivotal decision points such as the shift from implementing evidence-based strategies to adopting co-design approaches developed jointly with nurses and patients. At this stage of the analysis, insights were informed by Corry et al.’s model for the development of complex interventions [50]. Similarities and differences between studies were noted, particularly when variations in implementation processes did not align with person-centered nursing handover practices or with increased care safety, revealing gaps and areas for ongoing debate. In addition, we documented the influence of dominant author groups and research traditions, while observing underrepresented perspectives, such as that the patient has a role to play in NBH in reviewing safety aspects and self-managing their health, and that it is up to them to decide whether to take a more active or passive role in the handover and whether family members can participate.

After this familiarization, premises were formulated based on the work of dominant authors in the literature, identifying not only underrepresented points of view but also the factors contributing to the dominance of specific ways of thinking. From this analysis, three premises were generated to underpin the state-of-the-art understanding of patient participation during nursing handover: (1) patient participation in NBH is defined by distinctive attributes and purposes; (2) the way nurses conduct NBH is influenced by multiple factors that significantly shape the level of patient participation; and (3) the implementation of strategies designed to enhance patient participation in NBH leads to impacts that can be evaluated. Finally, these assumptions were evaluated by selected articles from the corpus to evaluate the completeness and soundness of the interpretations. Based on these three premises, the extracted data were synthesized and organized into three broad categories.

### 3.6. Reflexivity (Stage 6)

The analysis, synthesis, and interpretation of the corpus analysis were carried out by one researcher (P.C.) and subsequently confirmed by a second researcher (M.D.L.-F.). This analysis may have been informed by the post-positivist ontological assumption that nursing management is a contextualized social practice—not only rooted in individual patient and nurse situations but also in the ongoing structure of practice situations, where nurses influence and are influenced by each other’s practice [71]. It may also be informed by the epistemological assumption that nursing management synthesizes nursing science, management principles, behavior and organizational theories, and resource management to lead, manage, and design care systems appropriate to those ongoing structures of practice [72]. As part of the interpretation process, these assumptions were intentionally questioned by examining articles that contradicted this premise, in order to refine and adapt the findings from the literature.

## 4. Results

### 4.1. Studies Characteristics

**Figure 2:**
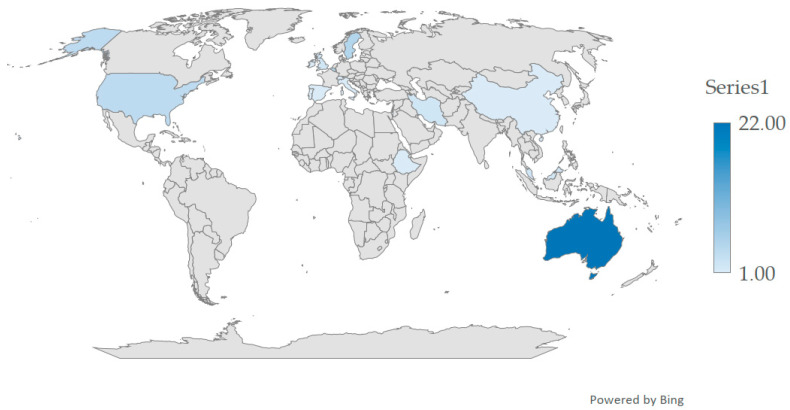
World map of the distribution of studies.

The authors analyzed a total of 50 articles, of which 36 were obtained from database searches and 14 through manual citation searching, all meeting the inclusion criteria. The included studies were conducted in various countries, including Belgium [73,74,75,76,77], Switzerland [78], Sweden [79,80,81,82,83], Portugal [84], Spain [85], Italy [86], Ireland [87], Israel [88], Iran [89,90], Ethiopia [91], Malaysia [7,92], China [93], Australia [94,95,96,97,98,99,100,101,102,103,104,105,106,107,108,109,110,111,112,113,114], the United States of America [25,115,116,117], and the United Kingdom [118,119]. Figure 2 displays the distribution of papers by country.

The studies were published between 1999 and 2025 and covered a wide range of clinical settings, including the following: medical–surgical inpatient units [7,25,73,74,75,77,78,79,83,84,87,88,90,91,92,94,95,96,98,100,101,108,109,110,112,114,117,118,120], critical care units [84,85,86,109,120], emergency departments [84,91], mental health settings [76,103,105,106,107,109,120], pediatrics [84,92,93], oncology units [80,82,99,101], obstetrics [25,83,84,92,110], palliative care units [101], rehabilitation units [73,74,75,98,102,108], operation rooms [84], delivery units [84], and dialysis units [108].

The review included 27 qualitative studies using a wide variety of methodological approaches such as semi-structured interviews [76,80,81,85,86,91,97,101,102,103,105,107,110,113,118,119], focus groups [7,76,94,102,103,112,115], questionnaires [95,99,116], discourse analysis [97,101], observations [97,99,101,102,104,118], field notes [97,104,118], and audio/video recordings of handovers [97,101,108]. Additionally, 17 quantitative studies were included, comprising quasi-experimental and experimental designs [25,74,78,83,89,90,93,106,117], observational studies [75,77], cross-sectional descriptive studies [79,92,96,100], longitudinal studies [82], and transcultural validation studies [84]. A smaller number of seven mixed-methods studies were also included [73,78,87,88,98,109,120].

Based on the studies included, the findings were categorized into domains of knowledge. These domains are defined as units of analysis representing the core of accumulated knowledge over a specific period, expressed as fields of study that have drawn the attention of research [121]. The review data were categorized into domains of knowledge to facilitate the establishment of research priorities and future investigation areas. The synthesis of findings was guided by the model of Corry et al. [50].

### 4.2. Domain of Distinctive Nature and Attributes of Patient Participation During the NBH

One of the domains identified in this SotA review includes the nature and distinctive attributes of patient participation in handover communication.

#### 4.2.1. Distinctive Nature of Patient Participation

In this review, it was found that patient participation in NBH has been used as a patient empowerment practice in the context of their self-care management capabilities and a patient information practice in the context of nursing care safety. The latter was highlighted in a study that explored the frequency and nature of patient participation in nursing handover, where NBH was recognized as a platform for information exchange that helps nurses better understand the patient and enhances patient safety [98], in addition to enabling patients to correct inaccurate information [105,118]. Moreover, this is also supported by the perspective of nurses, who noted that involving patients during NBH not only helps them understand the patient’s needs [92], but also engage with them in a structured review of safety aspects concerning their own care [80].

The informational nature of patient participation in NBH was also emphasized in the study by Paredes-Garza et al. [85], which described participation as a clarifying element for understanding a patient’s health status at the beginning of the shift. Similarly, in the study by Van de Velde et al. [76], both patients and health professionals affirmed that NBH should foster greater dialogue with nurses through the asking of questions and clarification requests, as well as through the definition of clear expectations and shared decision-making. The informational nature of NBH was highlighted in the study by Bradley et al. [111], where patients recognized the value of answering nurses’ questions and having the opportunity to ask their own.

In turn, the empowering nature of self-care management was highlighted in a study by Benham-Hutchins et al. [116], which assessed patient activation levels in managing their health after being hospitalized in a unit where nurses involved them in handover. The study found high activation levels in most patients. Similarly, the study by Ghosh et al. [120], revealed that involving patients in NBH increased their understanding of their health condition and empowered them to contribute to their own care. Additionally, the goal of enhancing patients’ self-care management capacities also emerged from the study by Paredes-Garza et al. [85], in which nurses recognized greater patient participation in NBH as a form of engagement in the recovery of their own health condition. This finding is also supported by Lu et al. [110], who reported that patients considered NBH a means to contribute important information regarding their own treatment and care regimen.

#### 4.2.2. Distinctive Attributes of Patient Participation

Alongside the nature of patient participation in handover communication, the key attributes of participation have been described in the scientific literature regarding NBH. These include the following: (a) inviting patients to participate in NBH [79]; (b) responding to nurses’ questions [79,111]; (c) listening to information shared by nurses [79]; (d) inviting patients to express themselves [79,111], to clarify their expectations and misunderstandings, and to share their opinions [112]; (e) encouraging patients to ask questions [94,96]; (f) asking patients to confirm or validate information [94]; (g) enabling patients to speak up and listen to nurses’ communication [79,96]; and (h) allowing patients to have a family member present during handover [96].

These attributes are based on the assumption that patients have a role to play during handover and that their participation in communication may have benefits for their care [105]. However, in a longitudinal study by Kullberg et al. [82], which investigated patient satisfaction with oncology care two years after NBH implementation, patients reported that the opportunity to see and hear nurse communication during handover did not necessarily mean they played an active role. In fact, in Van de Velde et al.’s study [76], patients expressed their desire to: (1) ask questions during NBH; (2) request clarification for unclear information; (3) define clear expectations; and (4) share in decision-making with nurses.

Furthermore, in a study by Benham-Hutchins et al. [115], most participants who had been hospitalized in wards that implemented NBH with patient involvement reported that, although they could participate in the handover communication, they did not have the opportunity to discuss their treatment plan or learn how to self-manage their health condition. In the study by Ghosh et al. [109], 18% of patients reported being unaware that they could participate in NBH or said they were “never” asked to do more than state their name and date of birth. In contrast, a study by Olasoji et al. [107], which explored the views of patients with mental illness about their experiences of being involved in nursing handover on an acute mental health inpatient unit following NBH implementation, found that patients felt their voices were heard through clarification requests and contributions to planning their own care. In the study by Hada et al. [94], nurses agreed that patient involvement in handover could increase their level of participation in care planning. This participation was observed in the study by Lantz et al. [83], which showed reciprocal communication during NBH and patient involvement in care planning.

However, the role that patients may play during handovers is subject to two key principles: respect for confidentiality and privacy of patient information. In the study by Bruton et al. [118], conducted in two acute wards with high patient turnover, the authors found that some patients were actively involved, others were less involved but wished to be more so, and some preferred to passively observe handover without participating. In some studies, patients explicitly stated that they should decide about their own participation and that of their family members in NBH [105,109], asserting their right to choose whether to participate and to consent to whether their information should be shared during that nursing practice, so as not to compromise their dignity and self-esteem [7]. Some patients noted that loss of privacy was a real issue [86,109] and that they preferred certain information (e.g., about mental state, mood, difficult conversations, etc.) to be shared only among nurses [105,110].

To mitigate the risk of breaches of confidentiality, since other patients in the ward might overhear the handover discussions, nurses in three mental health units of an Australian hospital—where NBH implementation was unsuccessful in terms of patient involvement—agreed that the information shared during NBH should be filtered by nurses [103]. This strategy was also emphasized by nurses in a study by Tobiano et al. [95], who worked in acute medical wards at a private hospital in Australia where NBH was implemented, by sharing sensitive information outside the patient’s room or in private settings. In the study by Whitty et al. [96], discussing sensitive matters away from patients was also considered crucial by the nurses. At the same time, Johnson and Cowin [112] reported that some nurses managed the discussion of confidential information flexibly within the NBH, even when they sought permission from patients and significant others beforehand.

### 4.3. Domain of Nurses’ Practices and Influencing Factors of Patient Participation During the NBH

Another domain identified in this SotA review encompasses nurses’ practices and the factors influencing patient participation in handover communication.

#### 4.3.1. Nurses’ Practices That Promote and Inhibit Patient Participation

The review found a variety of nursing practices, with different levels of patient involvement and participation, including the following: (a) practices where handover was conducted in the hallway [95,97,109]; (b) practices where handover was conducted in the patient’s room, and nurses directly invited the patients to participate [97,112,116]; (c) practices where handover took place in the room without involving the patient [91,97,111,116,117]; (d) practices where patients participated, for example, by making corrections, even though nurses did not involve them directly [97,116]; and (e) practices where nurses asked patients questions and responded to their concerns during handover [110,111,118].

Several studies also identified inhibitory practices, including the following: (a) positioning themselves at the doorway rather than entering the room [109,111,112]; (b) ignoring the presence of the patients [80,86,116]; (c) speaking in a low voice while looking at the handover sheet and avoiding eye contact [104,116,118]; (d) treating the patient like a number rather than a person [116]; (e) using medical jargon and acronyms [81,86,104,110,116]; (f) lack of time, concern about extending the handover duration, or appearing rushed [48,78,92,94,116]; (g) worries about the confidentiality of patient information [77,94,103]; and (h) the presence of many nurses during handover [94,95,116], particularly when positioned around the patient’s bed [80]. Regarding the number of nurses involved, patients tend to prefer that handover be conducted only between the outgoing and incoming nurses, rather than with the entire nursing team [96].

Some studies identified practices that promote patient participation. Bruton et al. [118] found that the communication style used by nurses during NBH influenced the level of patient engagement. Eggins and Slade [97] classified these styles as follows: (1) excluding the patient from NBH; (2) including the patient; (3) seeing the patient as a passive element; and (4) seeing the patient as an active element. Some authors noted differences between outgoing and incoming nurse styles. For instance, Forde et al. [87], in a hospital with one year of NBH implementation, observed that outgoing nurses seemed to limit patient participation, focusing instead on transmitting clinical data, assessments, and care plans. Similarly, Dahm et al. [104] noted that outgoing nurses did not greet patients, referred to them with impersonal third-person pronouns or terms of endearment, used jargon, ignored contributions from patients or accompanying family, and used task-oriented interruptions to end patient narratives or shorten the involvement of incoming nurses. Conversely, incoming nurses either actively engaged patients through introductions, first-name emphasis, collecting their input, and supporting contributions during NBH [104], or they passively received and processed information with little verification or clarification [87].

Other authors highlighted specific nurse behaviors that facilitate patient participation, such as inviting patients to participate in NBH [96,116]. In a study by Street et al. [98], which involved observing NBH and classifying patient behavior as (1) an active participant (asks questions, makes statements about their condition); (2) a passive participant (nods or makes short superficial comments); or (3) no participation (no form of patient communication during handover), facilitating practices were described as providing explanations during handover, making eye contact with the patient, asking the patient direct questions, asking patients for clarification, and giving patients or visitors the opportunity to ask questions. The study by Benham-Hutchins et al. [116] also found that nurses who acknowledged the value of patient contributions and spoke to patients rather than about them fostered greater involvement. Indeed, high scores obtained with the evaluation tool developed by Tobiano et al. [100]—in the dimensions of “conditions for patient participation in bedside handover” and “level of patient participation in handover”—led the authors to conclude that nurse behaviors promoting participation are a critical success factor for active patient involvement in NBH.

#### 4.3.2. Influencing Factors of Patient Participation

Regarding influencing factors, several studies reported that NBH was performed in hallways or with variable patient involvement [77,91,97,109,112,119], directly related to the care context [102]. In the study by Drach-Zahavy et al. [88], ward workload was negatively associated with both patient and nurse initiative during NBH. In addition, Dumbala et al. [91] found that patients and family members who were not intentionally involved in the handover were admitted to services lacking either an organizational handover policy or a clear description of the activities required for nurses to conduct an effective handover. Even in hospitals with policies supporting patient involvement and where handover was conducted at the bedside, patients demonstrated either passive or active behavior [98]. Other organizational and cultural context factors attributed to low patient participation include time constraints [7,78,80,109,117], patient acuity levels [7], lack of awareness that patients can play an active role in NBH [80], NBH being conducted when most patients were asleep [109], and the presence of family members [95,97]. Regarding the possibility of participation, in the study by Ghosh et al. [120], both patients and their families stated that knowing they could participate in NBH would have increased their confidence to ask questions during the handover.

The presence of family members was also identified by nurses as a factor influencing patient participation, due to concerns that their involvement could reduce the efficiency of the handover, with questions unrelated to the handover content [95]. However, in the study by Ghosh et al. [120], patients expressed that they would have liked family present when their condition prevented them from participating. In the same study, families who participated reported that their involvement helped identify and address key concerns, especially about post-discharge treatment planning, particularly for older patients, those with dementia, and non-English speakers [120]. In Wiklund et al.’s study [81], conducted in a maternity ward, partners were encouraged to actively participate in NBH when women who were postpartum were unable to do so due to pain. Drach-Zahavy et al. [88] also found that family presence was positively associated with both patient and nurse initiative during NBH. In addition to context-related factors, other patient-specific factors were identified, such as the patient’s clinical condition [95], patient preference [113,119], reasons related to infection prevention [97], the patient being asleep during handover [97], and the patient’s personality [88,114]. Regarding personality, a study by Drach-Zahavy et al. [88] in five surgical wards, which conceptualized patient participation in NBH as a bidirectional process involving both patient and nurse initiative, found the following: (1) neuroticism, extroversion, and conscientiousness traits were negatively associated with nurse initiative to promote patient participation in NBH; (2) neuroticism and agreeableness were positively associated with patient initiative in NHH; and (3) openness to experience was negatively associated with patient initiative.

Some nurse-related factors were also described. In a study exploring nurses’ opinions on the feasibility of involving patients in NBH, nurses attributed superficial patient involvement to a knowledge deficit and lack of experience in guiding nurse–patient interactions, limited use of therapeutic communication skills, and a task-oriented mindset [7]. Indeed, Lupieri et al. [86] reported that patients in an Italian cardiothoracic ICU felt the need to express emotions and concerns during NBH and wanted to be reassured, especially when clinical progress was unfavorable. Similarly, in the study by Yuen et al. [108], nurses showed low levels of encouraging emotional expression and accepting patient feelings during NBH. This aligns with the findings of Khuan et al. [7], where nurses admitted prioritizing physical assessment and patient comfort over psychosocial aspects when involving patients.

Regarding nurse-related factors, Eggins and Slade [97], in a unit with high patient turnover that had recently transitioned from traditional handover to NBH, observed a lack of confidence and communication skills among nurses for the new interactive context of bedside handover, possibly explaining the inconsistent patient participation. In fact, the study by Olasoji et al. [106], which implemented a training program targeting negative nurse attitudes and improving patient communication during handover, found a positive association between program effectiveness and improved therapeutic relationships with patients. That same study also identified additional nurse-related factors, namely, a negative association between intervention effectiveness and nurse age, and a positive association between effectiveness and years of professional experience in that specific setting [106].

### 4.4. Domain of Strategies and Impacts of Increasing Patient Participation During the NBH

In this review, we identified an additional domain related to strategies and impacts regarding patient participation in handover communication.

#### 4.4.1. Strategies for Increasing Patient Participation

Some studies suggested that increased patient involvement behaviors during NBH are associated with the use of protocols that standardize nurse behavior during handover. Malfait et al. [73,78] found that adopting information-focused handover protocols was associated with a reduction in handover duration, allowing nurses to engage more effectively with patients during the handover. They interpreted that promoting patient participation using these types of protocols could increase the overall handover duration. In the study by Abbaszade et al. [90], conducted in the coronary care units of two public hospitals in Iran that implemented SBAR in the NBH, it was concluded that this protocol can affect nurses’ performance by facilitating information exchange, reducing the possibility of physical loss of patient information during handover, and decreasing the communication load. In two studies by Ghosh et al. [109,120], inconsistent nurse behavior was reported regarding handover location and patient participation across all wards in two Australian hospitals that used information-transfer-only protocols (e.g., iSoBAR or SHARED). Unlike SBAR-type protocols [25], ISBAR [83,102], iSoBAR [120], ISBARR [75], and SHARED [120], only the CARE [101,102] and I PASS the BATON [89] protocols are specifically designed to actively involve patients in NBH.

In the study of Neamti et al. [89], which used a quasi-experimental design, statistically significant improvements in information quality, interaction/support, efficiency, and patient involvement were attributed to the use of the I PASS the BATON protocol. In Chien et al.’s study [102], nurses began using both ISBAR and CARE protocols to structure not only nurse–patient interactions but also the handover content. Furthermore, in the Ghosh et al. [109] study, 22% of patients suggested standardizing a handover process that includes patient and family involvement to improve practice consistency. Studies by Yang et al. [93] and Abt et al. [78] have shown that the use of handover protocols involving the patient allows for greater patient participation in NBH.

Active support from nurse managers in implementing NBH has been identified as a key strategy for encouraging nurses to adopt patient-involvement behaviors [83,98,101], even though managers themselves may require support to lead the implementation [103]. Studies addressing inconsistent patient involvement during NBH suggested that implementing this practice requires change management strategies led by nurse managers [25,101,103]. Drach-Zahavy et al. [88] found that the presence of nurse managers was positively associated with patient initiative during NBH. In a study by Lantz et al. [83], conducted in a hospital that adopted a patient-involvement policy during NBH, nurse managers were part of the implementation team that was training nurses on how to involve patients. As a result, patients showed increased participation in the following: (1) sharing their symptoms with staff; (2) reciprocal communication; (3) being told what was performed; and (4) taking part in planning. Similarly, Chien et al. [102] reported increased active patient involvement following a nurse manager-supervised intervention.

To adapt NBH to local contexts, several studies used co-design strategies involving nurses and patients jointly [73,76,99], nurses and nurse managers together [108], and nurses and patients individually [75,108]. These strategies helped identify preferred participation methods from the patient perspective [76] and clarified the purpose of patient involvement in NBH [76]. In Casey et al.’s study [99], a co-designed digital *App* enabled patients to communicate how they felt, any additional care needs, and health questions. It included a jargon dictionary and positively influenced patient participation, prompting nurses to address shared information during NBH and improving communication via the dictionary. Another study involving a digital *App* co-design for enhancing NBH communication found that 58% of patients asked questions during handover in addition to those submitted through the *App*: (1) greeting the patient warmly; (2) showing good nonverbal behavior; (3) allowing time for the patient to absorb information; (4) giving clear explanations; (5) involving the patient in decisions; and (6) exploring the acceptability of the care plan [108].

Regardless of whether implementation involves a protocol or co-design strategies, nurse manager involvement in change management is especially critical for ensuring consistent patient involvement. This was evident in the study by Malfait et al. [73], where a co-designed structured bedside handover protocol and education program—without nurse manager support—led to 30% of observed handovers not conducted at bedside and only partial patient involvement in one-third of completed handovers. Conversely, in Chien et al.’s study [102] involving a nurse manager from a rehabilitation ward in implementing and evaluating a tailored intervention using CARE and ISBAR protocols, handovers were successfully conducted at the bedside, and nurse–patient interaction during handover significantly increased.

Training was another strategy associated with greater patient participation during NBH. It helped to equip nurses to address confidentiality concerns, which are often cited as barriers. In Hada et al.’s study [94], nurses identified targeted education and clear guidelines as key to building confidence in handling sensitive information during handover. In another study that explored nurse and nurse manager perceptions of patient involvement in NBH, inconsistent nurse behavior was attributed to a lack of confidence and communication skills in determining what information to share [103]. A different study implementing a training module on handover processes, staff barriers, and supporting evidence found that training, combined with protocol use, significantly increased nurse compliance with patient involvement across medical–surgical and obstetric units [25]. In yet another study using training and co-developed recommendations tailored to the organizational and cultural context of seven hospital units, nurses began involving patients more actively in NBH without increasing handover duration [101].

To evaluate the success of implementation strategies, measurement tools are needed. This review identified two self-report instruments for measuring patient participation in NBH—one for patients [100] and another for nurses [84]. The patient-targeted tool was developed in Australia and assesses conditions for participation in bedside handover, his/her level of participation, and evaluation of participation outcomes. The nurse-targeted tool assesses direct engagement, personal interaction, information sharing, and their individualized approach. Patient participation conditions include individual patient attributes and their capacity to participate, as well as nurses’ interpersonal style, behaviors encouraging participation, and adaptation of information-sharing practices [100]. The level of patient participation concerns nurse–patient communication about care-related information (e.g., symptoms, capabilities, and regimens), encompassing both active participation (e.g., asking questions, responding to nurses) and passive participation (e.g., listening to nurse communication) [100]. Evaluation of participation in NBH addresses whether the patient’s expectations were met in terms of interaction quality, nurse–patient relationship, adaptation to the patient, comprehension, and satisfaction [100]. In turn, the nurse-targeted instrument evaluates direct engagement (how actively nurses encourage patient involvement); personal interaction (the extent to which trust is established during handover); information sharing (the degree to which information ensures patient safety); and individualized approach (how well nurses address patient-specific needs and preferences) [84]. These instruments’ dimensions stem from the refinement of the Bedside Handovers Attitudes and Behaviors scale [122], translated and validated in Portuguese, and a literature review [84]. The patient-directed instrument’s items were derived from theory and a systematic review [100]. The authors of both instruments reported strong evidence of the quality of psychometric properties (construct validity, content validity, and internal consistency), supporting their use within their respective cultural contexts [84,100].

#### 4.4.2. Impacts of Increasing Patient Participation

Some studies reported significant impacts of patient participation in handover communication. In the study by Kullberg et al. [82], patients who were encouraged to share their daily care experiences, ask questions, and speak up during NBH reported high levels of individualized care. Similarly, in a multicenter longitudinal study involving 13 units across five Belgian hospitals, statistically significant differences were found in the degree of individualization of care and in the level of patient participation in care as perceived by nurses [74]. Moreover, patient participation during NBH, even if passive, led patients [86,109], and accompanying family members [81], to experience a sense of safety and protection in relation to the care during the NBH [81,86,120], as well as a reduction in patients’ anxiety about their care [90,120]. This reassurance came from witnessing the complete and accurate transmission of information. This same sense of safety led patients in a mental health unit to identify knowing the responsible nurse for each shift as a beneficial feature of NBH [107]. In other studies, researchers reported that involving patients led them to perceive an improvement in the quality of care they received, resulting in more personalized and responsive treatment [113,120], and to report improved patient satisfaction with the exchange of information between nurses and patients. Table 1 summarizes the existing scientific evidence obtained and synthesized in this SotA review.

## 5. Discussion

This SotA review aimed to synthesize knowledge on patient participation during NBH and identify future directions for research and clinical practice. Next, we will discuss each domain of knowledge that emerged from this literature review. Subsequently, suggestions for future research will be presented that emerged from this review.

### 5.1. Domain of Distinctive Nature and Attributes of Patient Participation During the NBH

Understanding a problem that requires a complex intervention is one of the key elements of the model by Corry et al. [50]. To intervene in the inconsistency of patient participation during NBH, nurse managers need to understand the nature and distinctive attributes of patient involvement in NBH. In this SotA review, a tendency to implement NBH as a practice that is simultaneously empowering and informational was found. This dual nature of NBH practices is consistent with the findings of Pun et al., who observed improvements in the frequency of interactions as well as the quality and completeness of patient information presented during handovers after implementing training for nurses on the use of the CARE protocol [123,124,125]. In fact, in any type of handover, there is an informational dimension that allows healthcare professionals to gain as complete a situational awareness of each patient as possible [126], which can be further enhanced by the interactive dimension. Situational awareness is considered a fundamental competency that contributes to patient safety outcomes and high-quality inpatient care [127], and it is a product of monitoring patients’ conditions [128]. According to Stanson et al. [129,130], who proposed the theory of Distributed Situation Awareness, situational awareness arises from interactions between healthcare professionals, as well as from their interaction with the environment and individually held schemata. Each healthcare professional interprets the situation based on their individual experience, available data, and different types of situational awareness. Situational awareness is defined as the perception of patient data by healthcare professionals in a given space and time, the understanding of its meaning, and the projection of the patient’s future status, which may require essential nursing interventions [131]. A conceptual analysis conducted by Sitterding et al. [132], which aimed to define and analyze the concept of situational awareness in nurses’ work, identified additional attributes, including the following: (1) knowledge and expertise; (2) cognitive overload; (3) interruption management; (4) task management; (5) instantaneous learning; and (6) cognitive stacking.

Situational awareness is influenced by fatigue, nursing experience, team communication, nurse alignment, care situations, shift timing, and data visualization [127], but also by patients’ and families’ stories [133]. Additionally, it is an internal factor in nurses’ decision-making, playing a critical role in missed nursing care [134]. It is, therefore, considered a precursor of omission errors and preventable adverse events [131]. When shared, for example, with other nurses or with patients and families, situational awareness becomes a critical team competency, guiding effective action coordination. It is the only way for diffuse decisions made outside of the handover to remain consistent [133], and it is a highly relevant factor for developing person-centeredness in healthcare services [135]. According to Keefe et al. [136], situational awareness corresponds to the first level of handover, followed by level II (understanding) and level III (projecting situational awareness), across all areas: (a) patient status; (b) team; (c) environment; and (d) progress. O’Keefe et al. [136] argued that a good handover requires incoming nurses to have the opportunity to understand the situational awareness and mental models of outgoing nurses and to ask questions if their understanding does not align. Constructing mental models of the situation is essential for nurses to have an updated understanding of patients’ conditions [137]. A study conducted by Galatzan et al. [138] found that bedside handovers contained more information and less data, whereas non-bedside handovers contained more data and less information. This suggests that NBHs may better support the construction of mental models than non-NBHs.

In turn, this empowering nature is consistent with studies reporting increased patient autonomy and effective information sharing among nurses, patients, and the family members present [34]. This aspect is further supported by the findings of Tobiano et al. [65], who explored perceptions of non-participation among patients and hospital nurses, concluding that patients who were not involved in NBH did not participate in self-care at the time of discharge or during hospitalization. Additionally, a scoping review [45] highlighted that promoting self-care management competencies related to patients’ clinical condition is an important activity to promote patient involvement during NBH. This review identified the nurses’ adaptation of patients’ self-management of hospital activities as a strategy to promote patient engagement during hospitalization, including patients performing specific self-care activities such as monitoring temperature, symptoms, and fluid balance, self-administering medications under supervision, performing activities of daily living such as mobility and/or continence, and promoting recovery. According to Riegel et al. [139], self-care maintenance and monitoring activities are essential for developing patients’ capacity for self-care management of healthcare needs. Self-care management of healthcare needs is defined as the evaluation of symptom recognition, the importance of changes in signs and symptoms, and the effectiveness of a particular treatment [140]. Furthermore, self-management of healthcare needs is a care model that recognizes the patient’s primary role in taking responsibility for health promotion, disease prevention, and managing their clinical condition [141]. To develop these competencies, patients must be aware of their clinical condition [140]. A mixed-methods study by Pollack et al. [142], which analyzed team-based situational awareness, found that patients and families developed awareness of their clinical condition simply by participating passively in nursing handover. In addition, the repetition of critical information across multiple handovers offered patients and families opportunities (1) to hear different perspectives from various nurses with different backgrounds and specialties, and (2) to interact with that information [142].

Analysis of practices that influence the problem being addressed, as shaped by the scope of nursing practice, is another important element in Corry et al.’s model [50]. Practice analysis is defined as a tool for managing workplace change [143] that allows identification of activities in specific nursing practice contexts [144]. These activities are essential for identifying, in practice, the characteristics or attributes of a given concept [145]. In this review, we listed attributes that have been used to characterize patient participation during NBH from both the nurses’ and patients’ perspectives. These attributes are consistent with the concept of Person-Centered Handover Practices proposed by de Lange et al. [146], for all clinical, intra- and inter-disciplinary handover practices that include the patient and/or family at the bedside. They are also aligned with Schuster and Nykolyn’s [147] concept of Safe Communication, defined as the activities of gathering and sharing information, and clarifying and verifying the accuracy of interpretations made, carried out by nurses collaboratively with patients, their families, and other healthcare professionals, in pursuit of common goals related to the provision of safe, high-quality care. In addition, they are supported by the studies of Bårdsgjerde et al. [148,149], who proposed interaction as one of the elements of the Comprehensive Model for Patient Participation, which includes the following: (a) patients’ contribution to the direction of action (e.g., through initiation or response); (b) patients’ influence in defining the problem; (c) patients’ role in the reasoning process (e.g., discussing the issue and possible solutions); (d) patients’ influence in decision-making; and (e) emotional reciprocity between patients and health professionals. The notion that patients have a role to play during handovers and that their participation can benefit their care aligns with the levels of involvement defined in this model, which supports different forms of participation—from active to passive. The model includes the following: (a) non-involvement; (b) information seeking/receiving; (c) giving information/dialogue; (d) shared decision-making; and (e) autonomous decision-making [148,149,150].

The dependence of the patient’s role in NBH on the principles of confidentiality and privacy in promoting participation is grounded in the ethical duty of nurses, as outlined in the codes of ethics by the American Nurses Association and the International Council of Nurses [151,152], to promote, advocate, and protect patients’ rights, health, safety, and interests in the use and disclosure of personal information. Regarding confidentiality, the American Nurses Association [151] states that patients’ rights to confidentiality and privacy are the main factors that nurses must consider when making decisions about using or sharing patient information. Disclosure for purposes such as continuity of care, quality improvement, and risk management is permitted if (1) defined in institutional policies, mandates, or protocols; (2) the use of information is relevant to the activity; and (3) the patient’s rights, safety, and well-being remain protected [151]. Confidentiality is defined as the non-disclosure of personal information without the patient’s express permission, while privacy is the right to control access to one’s own information, including when, how, and to what extent that information is shared [151]. The International Council of Nurses defines personal information as any information obtained during professional contact between nurses and patients/families that, if disclosed, may violate privacy or cause distress, embarrassment, or harm [152]. Despite the duty to respect patient confidentiality and privacy, Chien et al. [101] argue that bedside handover is a way to recognize all patient rights, particularly the right to participate in their care through active involvement. To engage in handover communication and, consequently, in their own care, nurses must also respect patients’ right to know or not to know their care plan [153]. In the study by Ghosh et al. [109], some patients emphasized the importance of nurses informing them and their families about their right to participate in handover communication. The right to participate is commonly referred to as the “right of rights” [154].

In this domain, a tendency was observed to describe patient participation in NBH as a practice that is both informational and empowering, based on the premise that the patient has an important role, the defining characteristics of which are not yet adequately systematized in the scientific literature. This dual informational and empowering nature of patient participation in handover communication has not yet been robustly explored in handover research, neither empirically nor theoretically.

### 5.2. Domain of Nurses’ Practices and Influencing Factors of Patient Participation During the NBH

Solving a problem through a complex intervention involves both conceptual and empirical work to understand the issue, not only at a theoretical level but also operationally through its manifestations [155]. Within the practice analysis element of the Corry et al. [50] model, we identified a set of nursing practices showing different levels of patient involvement and participation, in which nurses may exhibit behaviors that either promote or inhibit patient participation during NBH. Nursing practices can be defined as the set of cognitive, behavioral, and social aspects of professional actions performed by nurses to meet patients’ needs and fulfill their roles, including how nurses think, make decisions, transfer and apply knowledge in specific situations, or perform specific actions [156]. The variability in nursing practices aligns with findings from some studies showing that NBH is carried out differently than designed [157], and that it is not always conducted or may involve just greeting the patient. A mixed-methods systematic review by Tobiano et al. [52], which synthesized nurse practices related to patient participation in NBH into nurse-centered and patient-centered handover practices, also supports the variability we described in this study. The different behaviors of nurses and patients are also consistent with findings from a qualitative evidence systematic review by McCloskey et al. [38], which reported, as a synthesized finding from the experiences of patients and nurses, the variation in desire and ability to participate in NBH. The same authors revealed in this review that nurses use adaptive practices to address patient-related, family-related, and environmental factors influencing NBH, which manifest in a variety of practices [38]. The diversity of practices identified is consistent with the results of a systematic review by Buus et al. [49], who reported that nurses adopt an adaptive and flexible approach during NBH according to the following: (1) the context of the patients’ clinical situation; and (2) the needs of the incoming nurses at shift change.

Beyond understanding the practices demonstrated by nurses, a problem that requires a complex intervention also involves understanding the influencing factors [158]. This understanding is essential because every new intervention involves a behavior change in its actors [51]. In this SotA review, we described three levels of factors influencing patient participation during NBH: (1) factors related to the care context; (2) factors related to the patients; and (3) factors related to the nurses. These three levels of factors align with some categories included in the conceptual framework proposed by Maher et al. [34]. Forde et al. [40], in an integrative review aimed at identifying factors to be considered in NBH studies, classified the factors as related to the following: (1) who (persons involved, staffing levels, the person during handover, and demographics); (2) where (location of handover, details of patient’s room); (3) how (teamwork, handover process, use of structured communication tools, communication process, and information volume and complexity); (4) when (timing, duration, and types of interruptions); and (5) what (identification, situation, background, assessment, recommendations, risk, read-back, patient education, social conversation, consideration of system-level issues, and articulation of care transfer). A study conducted by Alrajhi et al. [159] identified environmental factors as a distinct category from those adopted in other studies. Cruchinho et al. [60] referred to environmental factors as “factors related to the care setting”. Among these environmental factors, we highlight the presence of family members during NBH. This factor was also described by Malfait et al. [160] alongside others, including the following: (1) handover duration; (2) lack of ability/skills to partner with patients/share power/patient participation; (3) confidentiality/privacy; (4) nurse–physician relations; (5) patient’s competence indicators; (6) nurses’ perceptions regarding handover; (7) nurse manager’s role; (8) hospital processes; (9) structured handover; (10) loss of socializing, overview, and collegiality among nurses; (11) role of colleagues; and (12) visiting times/family presence. Contrary to Malfait et al.’s findings [160], in our study, the presence of visitors was identified as a factor promoting patient participation.

In the context of Corry et al.’s model [50], knowledge of the diversity of patient involvement practices in handover, as well as the factors that influence the occurrence of these practices, allows researchers not only to identify the problem that requires intervention but also its overall objective and goal. In this domain, a research trend was observed toward identifying nurses’ practices either as barriers or as facilitators of patient participation in NBH, based on the care context, nurses, and patients influencing factors. However, we did not find studies that explore, using examples of good practices promoting patient participation in NBH, the knowledge, skills, and attitudes nurses require to conduct bedside handover with the involvement of patients and families through communication. We also did not find studies that explore how outgoing and incoming nurses seek to involve patients in the handover process. These studies are necessary for guiding nurse managers in supporting the factors that influence nursing practices in the processes of implementation and improvement of NBH [25].

### 5.3. Domain of Strategies and Impacts of Increasing Patient Participation During the NBH

This review showed patient participation in NBH with the use of handover protocols that include both interactional and informational elements. An integrative review of the literature by Anderson et al. [42], focusing on the implementation of NBH, reported the exclusive use of handover protocols without incorporating elements of patient involvement. Similarly, a systematic review by Cho et al. [161], which examined the effects of quality improvement (QI) projects on NBH, found that patient and family involvement was limited to handover protocols containing informative elements. In general, handover protocols help increase teamwork during shift transitions within a unit [162]. Regarding information-focused protocols, some authors suggest that their use reduces the likelihood of important information being omitted and prevents critical information from being subject to cognitive bias and interpretation problems by different team members [163]. They also ensure awareness of the situation among the different actors involved in the handover and allow teams to implement the information they consider necessary during this practice, thereby optimizing the amount of information transmitted [164]. Although there is considerable evidence supporting the idea that these tools improve handover, their use can have different effects depending on the following: (1) how the tool is integrated into the team; (2) how nurses are trained; and (3) the usability of the tool itself [165]. When using a handover protocol, the content transmitted during handover can vary depending on the knowledge, experience, and individual style of the outgoing nurse. Some nurses provide excessive information, while others offer minimal or low-quality information [94]. Despite valuing the standardization of handover processes, some authors argue for a flexible approach rather than a rigid structure [166].

Regarding strategies, we discovered two handover protocols that increase patient participation in NBH: I PASS the BATON and CARE. In a study conducted in several hospitals, Thomas and Donohue-Porter [167] implemented the I PASS the BATON protocol, which cues nurses on the information to include in handover. This intervention led to improvements in patient satisfaction across three domains: “nurses kept you informed” (4th percentile to 67th percentile); “friendliness and courtesy of staff” (19th percentile to 92nd percentile); and “likelihood to recommend” (49th percentile to 61st percentile). The use of this type of tool is consistent with the findings of Street et al. [168], who have found that the use of standardized handover protocols increases patient engagement in NBH. Moyo et al. [169], in an exploratory review synthesizing nurses’ experiences and perceptions of implementing structured clinical handover frameworks, recommended adding a letter in the handover protocol, representing patient involvement and serving as a reminder for the team to actively involve patients in their clinical handovers.

The I PASS the BATON protocol is part of the TeamSTEPPS^®^ program [170], and is considered suitable for NBH because, in addition to information, it includes an element aimed at involving the patient in the handover (introduction, patient, assessment, situation, safety concerns, background, actions, timing, ownership, and next). Patient involvement occurs at the end, for example, by asking about their current concerns [167]. Adopting the I PASS the BATON allows for checking whether nurses are consistently involving patients in NBH and whether patients are routinely active participants in handover [171]. The Connect, Ask, Respond, and Empathize (CARE) Protocol, linked to ISBAR, allows nurses to deliver accurate, concise, and logically structured information while promoting patient participation [172]. The acronym stands for Connect, Ask, Respond, and Empathize [124,172]. A scoping review by de Lange et al. [173] highlighted that handover protocols are important for guiding nursing practices but must be context- and patient-specific. Thus, each context should decide whether to use I PASS the BATON or the CARE protocol in NBH.

In this review, the role of the nurse manager was identified as a key strategy in implementing and sustaining NBH, given that ensuring consistent promotion of patient participation in NBH is a significant challenge for nurse managers [174]. This finding is supported by Brown-Deveaux and Gabe [171], who reported that, for the redesign of the NBH process to positively affect patient experience across pilot units, it was necessary to appoint specific nurse leaders for each unit, supervised by nurse managers. According to Sidadi and Braden [175], when designing a complex intervention, it is important to consider influencing factors by involving various stakeholder groups (patients, healthcare professionals, managers, and others), since their contributions are valuable for identifying contextual factors and how they can be addressed to adapt the intervention to specific characteristics and resources. When evaluating intervention implementation, it is also important to monitor these factors and determine how they affect implementation [175]. This requires nurse managers to possess change management skills, that is, the ability to prepare healthcare environments for transitions to new structures, processes, or outcomes [176].

Change management is defined as a systematic and structured process of developing and implementing strategies and interventions to help healthcare organizations transition from a current to a desired state [177]. One of the attributes nurse managers use in change management involves gaining insights through detailed information collection that is contextually analyzed and interpreted. With this information, nurse managers aim to understand the organizational context, identify problems, develop change strategies, and evaluate the outcomes [178]. According to Harrison [179], to lead change processes, nurse managers should be trained in Implementation Science, Improvement Science, and Strategic Change Management—areas also considered essential by the American Nurses Association [180].

Two evaluation instruments specific to patient participation in NBH were also identified—one assessing nurses’ perspectives and the other patients’ perspectives. This finding is consistent with an integrative review by Forde et al. [40], which argued for the need for specific tools to comprehensively study and understand the complexities of NBH implementation. Today, the patient’s perspective is seen as a valuable source of knowledge about healthcare and is central to continuous quality improvement programs [181]. Although healthcare has traditionally neglected measuring the impact of its activities on reported outcomes and experiences, systematically collecting this information is essential for the transformation toward people-centered health systems [182]. One way to evaluate patients’ experiences and needs is through Patient-Reported Outcome Measures (PROMs) and Patient-Reported Experience Measures (PREMs).

PROMs and PREMs are questionnaires used to measure patients’ perceptions of service outcomes and experiences [183]. PROM is an umbrella term covering all subjective outcomes reported by patients without healthcare professional interpretation [184], and can be applied before, during, and after care [185]. PREMs measure respondents’ perceptions of provided services and are considered process measures rather than outcome measures [185]. Clinician-Reported Outcome Measures (CROMs) are assessments reported by healthcare professionals that include a wide range of methods such as observations, performance tests, and measurements [186]. The availability of both a PROM and a CROM focused on patient participation in NBH allows future research comparing nurse and patient perspectives. Used together, they enable nurse managers to perform more rigorous evaluations of patient participation in NBH. Moreover, they support patient-mediated interventions—a type of complex intervention [187], aimed at changing nurses’ behaviors through patient interaction and assessing the impact of such change on outcomes [188].

Co-design emerged in this review as a valued strategy for improving patient participation in NBH. This is consistent with the development of interventions in other types of handover [189] and other areas of intervention to increase patient participation [189]. Co-design involves meaningful engagement of end users at any stage of the research or quality improvement process—from planning to dissemination [190]. Co-design can be used to improve healthcare, develop planning documents or specific policies, and enhance governance, education, or tools [191], and is considered useful for designing and evaluating complex interventions related to patient participation [192]. This method has also been renamed experience-based co-design to emphasize the importance of involving staff and patients in designing health services [193]. It often uses qualitative methods and tools such as focus groups, affinity diagrams, open, semi-structured, or closed interviews, creativity methods, observation, life stories, and shadowing, though it does not exclude measurement and quantification [194].

The methods used in co-design should consider the characteristics of the clinical context. For instance, Bonaconsa et al. [195], in a study developing a handover tool for optimizing infection control and antimicrobial stewardship in an intensive care unit, used interactive journal clubs to address nurses’ limited availability for group activities due to time constraints and high patient acuity. A common critique of co-design is that it is sometimes conducted to justify pre-existing strategies and concepts rather than open healthcare systems to new perspectives and practices [196]. To avoid this, it is recommended that nurse managers (1) ensure all partners contribute meaningfully to decision-making; (2) establish clear roles and responsibilities; (3) clarify the project’s value and purpose; (4) set realistic expectations; (5) value all contributions; (6) integrate critical reflection; and (7) shift power [197]. To our knowledge, there is limited research using co-design methods involving both nurses and patients. Since nursing management focuses on designing, facilitating, supervising, and evaluating care delivery systems [180], improving NBH offers an excellent opportunity for nurse managers and researchers to engage patients in co-designing the handover process.

This review also found that nurse training is a key strategy for increasing patient involvement in NBH. Yusrawati et al. [198], in a study evaluating a training program including information about bedside handover, hospital policies, and demonstrations or role-playing, concluded that training effectively increases nurses’ knowledge, attitudes, and skills. Choi et al. [199], in a systematic review of educational interventions for improving NBH, found that demonstrations, individual feedback, and hands-on practice effectively improve nurse performance.

To enhance patient participation in NBH, training programs should help nurses develop communication skills for using protocols with interactional elements. For example, Eggins and Slade [172] created the “Better Bedside Handover” face-to-face training program and a train-the-trainer package, including the CARE and ISBAR protocols. The training included the following: (1) creating engagement (with reviews on poor handover risks, the role of communication, benefits of bedside handover, and addressing nurses’ reservations); (2) self-reflection using video reconstructions of NBH; (3) strategy and protocol input; and (4) role-play activities using real patient scenarios [172].

Training programs that include protocols combining informational and interactional elements seem to effectively increase patient participation in NBH. Eggins and Slade linked ISBAR to CARE and reported that training significantly changed nurse behaviors in patient engagement [172]. Abt et al. [78] also reported a training program incorporating the I PASS the BATON protocol, role-playing, clinical vignettes, and simulations involving actors, among others, composed of two sessions and a total of seven hours. The first session aimed to describe the basic elements of bedside nursing handovers, to develop handovers inspired by clinical cases encountered on wards according to the principles of I PASS, and to share experiences related to this change in practice. The second session aimed to recall the essential elements of high-quality caregiver–patient relationships, to perform a standardized patient–nurse bedside handover process in a complex care context, to state elements that could slow the implementation of this approach by providing observations made in simulations and personal experiences, and to reinforce positive drivers for implementing this new practice [78]. After training, some barriers remained, including time constraints, confidentiality concerns, and patient interruptions [78].

Finally, this SotA review identified patient participation impacts, focusing mainly on patients and the nursing care process. At the care process level, we found studies addressing individualized care impacts. This aligns with the notion that the ability to provide individualized care depends on knowledge of the patient’s individuality [200], which can be obtained through NBH. Individualized care reflects nurses’ attention to patients’ needs, preferences, behaviors, feelings, perceptions, and understanding, influenced by nurse characteristics (e.g., academic training, being a specialist, age, professional experience, personal motivation, empathy, and culture), and organizational factors (e.g., staff ratio, care routine and standardization, autonomous professional practice, leadership, and positive work environment) [201]. This impact is also supported by Bressan et al. [19], who in a qualitative meta-synthesis found “being the center of nursing care processes” as a central patient experience theme. At the patient level, we described impacts on perceived safety. This aligns with findings from Yan et al. [202], who identified patient participation as a facilitator of perceived safety. Maintaining a safe environment is a basic survival need for patients and includes internal biological/physiological factors and the external surroundings [203]. To ensure safety during hospitalization, nurses need secure communication skills to (1) transmit, gather, and exchange sufficient information for shared understanding; (2) accurately convey and interpret messages; (3) unambiguously express verbal messages; (4) adapt to environmental challenges like noise or time pressure; and (5) respond to expressed needs and expectations [204]. We also identified patient satisfaction as an impact of participation in NBH. This is consistent with findings from White-Trevino et al. [205], who reported satisfaction with structured handover processes when patients were involved in nurse communication.

In this domain, there is a trend for nurse managers to take an active role in managing the change toward NBH, both in designing interventions addressing nursing practices and in directly training and supervising nurses. There is also a tendency to combine co-designed interventions, nurse training strategies, and the use of handover protocols with informational and interactional elements to guide nursing practices. Despite the limited number of studies describing NBH’s impact on care processes and patient outcomes, no studies were found that assessed process measures reported by patients that could enable the assessment of “Patient Safety”.

### 5.4. Patient Participation in NBH as a Complex Intervention

Patient participation in NBH is a complex intervention [77]. Smeuleurs and Vermeulen [206] justified this classification because communication during NBH has a strong interactional component and depends on the care context in which it is performed. In this SotA review, we adopted Corry et al.’s [50] complex intervention development model, as it provides a framework for nurse managers to design change management interventions within their professional scope. Considering that nursing management is devoted to the design, facilitation, supervision, and evaluation of nursing care systems [180], it becomes clear that the ability to design change management interventions is an essential skill for nurse managers that should be included in their academic preparation. These types of interventions are organizational in nature [207] and are defined as a set of intentional activities designed to facilitate planned organizational change by influencing the response of recipients and the adoption of a change project [208]. They may take various forms, namely, (1) communication (informing, framing, and dialogic); (2) support (training, coaching, and organizational change support); (3) involvement (consulting, co-creating, and co-deciding); (4) reinforcement (rewards and goal-setting); (5) social influence (role modeling and peer exchange), and (6) coercion [208]. Regardless of the form, the first stage of change management intervention aims to diagnose the problem or identify the opportunity for change [209].

The synthesis of empirical evidence from this SotA review provides nurse managers with relevant knowledge for the development of change management interventions. The results within the Domain of Distinctive Nature and Attributes of Patient Participation during NBH and the Domain of Nursing Practices and Factors Influencing Patient Participation during NBH help nurses identify performance gaps relative to standards, define the overall goals of the intervention, and determine the activities to be implemented. These domains also support the drafting of intervention components—such as implementing a specific handover protocol or a targeted training workshop—and the selection of appropriate theoretical frameworks and change management processes for intervention delivery. In turn, insights from the domain of strategies and impacts of increasing patient participation during the NBH allow not only the development of a method for designing and refining complex interventions [74], and the formulation of their components, but also the definition of processes for evaluating outcomes using PREMs, PROMs, and/or CROMs.

### 5.5. Future Directions in Research

These findings improve nurse researchers’ understanding of patient participation during NBH and provide new insights for the development of complex interventions that aim to increase this participation. In the domain of distinctive nature and attributes, it is necessary to continue exploring the empowering and informational nature of promoting patient participation in NBH, refining existing theories on patient participation in care. This refinement will enable the development of theory-driven studies in the implementation or improvement of NBH, particularly through complex interventions aimed at engaging patients and families during the handover process.

Studies are also needed to assess the impact of patient involvement during NBH on patients’ self-management capabilities regarding their clinical condition. Although we have described the distinctive attributes of the concept of patient participation in NBH, this alone is not sufficient to define the concept of patient participation in NBH both theoretically and operationally, nor to outline its antecedents and consequences. Therefore, concept-analysis studies are needed—both comprehensive and in-depth—on the use of the concept of patient participation in NBH, considering the different levels of involvement and participation in communication. This type of study may involve the creation of model cases, which could be used in nurse training design and in building complex interventions to enhance patient participation.

In the domain of practices and influencing factors, we identified a need for studies, such as focus groups centered on good practice examples, that explore the knowledge, skills, and attitudes behind nursing practices that promote patient participation in handover communication. Grounded Theory studies are also needed to deeply understand how nurses foster patient participation in NBH, whether at the end of their shifts or when starting a new one. Moreover, given the importance of nurses’ promotional practices in encouraging active patient participation in NBH, and the need to consider limiting factors when planning change management interventions, it is also essential to review and systematize the factors that, from the nurses’ perspective, influence patient involvement and participation in NBH communication. This systematization could be valuable for designing complex interventions aimed at increasing patient participation in NBH. Furthermore, additional studies involving the presence of family members during NBH are needed, as well as studies focused on improving communication skills for the interactional context.

Lastly, in the domain of strategies and impacts, more studies are needed that use handover protocols with both informational and interactional components, as well as interventional studies involving active participation of nurse managers in the processes of implementation and maintenance of change. These should evaluate the effects of targeted interventions co-designed with the involvement of nurses, nurse managers, and both nurses and patients. Further studies are also needed to equip nurses with communication skills specific to nursing handovers, especially for using protocols that integrate both informational and interactional components. These studies should include tools for evaluating patient participation in NBH. Regarding the impacts of patient participation in handover communication, interventional studies are required that focus on measuring the impact of increased patient participation in NBH on indicators of nursing care quality and safety, particularly those based on processes and reported by patients themselves.

### 5.6. Study Strengths and Limitations

As in all SotA reviews, it is important to understand how current knowledge has been achieved. We consider that the collection of contextual information regarding the settings of the studies included is a strength of this review. Another strength lies in the clear identification of research trends within each domain of knowledge under the broader research topic. The categorization of study data into knowledge domains facilitated the synthesis of current understanding and the identification of future directions for bedside handover research. The Model for Developing Complex Nursing Interventions guided the researchers in identifying data relevant to nurse managers. Additionally, the assessment of the methodological quality of the included studies is also a strength of this review. However, the high variability in study designs, sample sizes, and cultural contexts of the studies included is a limitation to be considered when generalizing the results. Another potential limitation was the exclusion of gray literature from the eligibility criteria, as only peer-reviewed published studies were included.

## 6. Implications for Comprehensive Care and Health Services

Comprehensive care is defined as care that simultaneously includes physical and mental health, wellness, prevention, and illness care [210]. It represents the primary goal of nursing care delivery [211]. To develop this approach, healthcare organizations and professionals need, among other things, to promote a culture of safety and foster partnerships with patients and families [212]. The implementation of NBH with the involvement and participation of patients in handover communication presents an opportunity to promote comprehensive care within healthcare organizations. The findings from this SotA review across the three domains of knowledge can contribute to the development of this approach in healthcare services and institutions. In the domain of the distinctive nature and attributes of patient participation during NBH, the findings enable researchers to improve the diagnosis of inconsistencies in nursing practice during NBH, which they aim to address, as well as clarify the objective of implementing or improving NBH interventions. In turn, findings in the domain of nurses’ practices and factors influencing patient participation during NBH allow researchers to use this information to develop targeted interventions, particularly for planning training programs for nurses. Finally, the findings in the domain of strategies and impacts of increasing patient participation during NBH can support researchers and nurse managers in identifying effective strategies to enhance patient participation in NBH, as well as guide the evaluation of the planned intervention.

## 7. Conclusions

In this review, it was found that patient participation in NBH has a dual nature: it is both empowering, in the context of enhancing patients’ self-care management abilities, and informational, in the context of nursing care safety. The main attributes of patient participation in NBH were identified, which are based on the requirement that patients have a role to play during handover—one that can benefit their care—and are governed by two key principles: respect for confidentiality and privacy of patient information. It was also found that nursing practices show varying levels of patient involvement and participation, and we systematized both the inhibiting and promoting practices related to patient participation in NBH communication. We described the factors that may explain the diversity in nursing practices, classifying them into those related to the care context, the patient, and the nurses themselves. Among the contextual factors that were highlighted, the presence of family members during NBH and among nurse-related factors emphasized the lack of confidence and communication skills needed for interactional contexts. The importance of using handover protocols that incorporate both informational and interactional components was underscored and identified the role of nurse manager as a key strategy for implementing and sustaining NBH. We also noted a growing trend toward the use of co-design strategies, involving primarily nurses, nurse managers, and patients in the development of targeted interventions. Training nurses is a fundamental strategy for increasing patient engagement in NBH, and consequently, their participation. Two new instruments for measuring patient participation in NBH were described, one designed for nurses and one for patients. Additionally, some impacts of patient participation in handover communication were outlined at both the patient level and the care process level within the organizational context. This review contributes to the advancement of nursing knowledge not only by providing an original synthesis that had not yet been conducted, but more importantly, by interpreting the results from the perspective of those who must act to improve patient participation in handover—namely, in the context of developing and implementing complex interventions by nurses.

## Figures and Tables

**Table 1:** Summary of existing scientific evidence.

Domain of Knowledge	Description
Domain of Distinctive Nature and Attributes of Patient Participation during the NBH	**Distinctive Nature of Patient Participation** Patient participation in NBH has been used not only as a practice for informing patients within the context of nursing care safety but also as a means of patient empowerment, supporting the development of self-management skills in preparation for discharge.
	**Distinctive Attributes of Patient Participation** NBH is characterized by attributes grounded in the recognition that patients have an active role, while respecting confidentiality and privacy. Patient participation challenges nurses to manage sensitive information appropriately.
Domain of Nurses’ Practices and Influencing Factors of Patient Participation during the NBH	**Nurses’ Practices that Promote or Inhibit Patient Participation** NBH involves a variety of nursing practices with different levels of patient involvement and participation. Nurses may demonstrate behaviors that either promote or inhibit patient participation, depending on whether they are starting or ending their shift, influencing the behaviors adopted by patients who are able and willing to participate.
	**Influencing Factors of Patient Participation** Patient participation in NBH is influenced by contextual factors (e.g., organizational handover policies), patient-related factors (e.g., clinical condition or being asleep during NBH), and nurse-related factors (e.g., limited use of therapeutic communication skills).
Domain of Strategies and Impacts of Increasing Patient Participation during the NBH	**Strategies for Increasing Patient Participation** NBH implementation strategies, such as the use of co-design approaches to interventions, the adoption of handover protocols to standardize nurses’ performance during handover, active support from nurse managers in change processes, and nurses’ training, are associated with increased patient participation.
	**Impacts of Increasing Patient Participation** Enhanced patient participation in NBH affects nurses’ perceptions of individualized care provided to patients, including safety and protection, reduces patient anxiety, improves patient satisfaction with information exchange, and positively influences perceived quality of care.

## Data Availability

Data is contained within the article or Supplementary Materials.

## Supplementary Materials

The following supporting information can be downloaded at https://www.mdpi.com/article/10.3390/nursrep15120438/s1; Table S1: Scale for the assessment of narrative review articles [213]; Table S2: Summary of included papers and the appraisal quality obtained.

## Public Involvement Statement

No public involvement in any aspect of this research.

## Guidelines and Standards Statement

This manuscript was draft against the Scale for the Assessment of Narrative Review Articles (SANRA)—https://www.aerzteblatt.de/down.asp?id=22861 (accessed on 13 June 2025) to report this SotA Review.

## Abbreviations

The following abbreviations are used in this manuscript:

CINAHL	Cumulative Index to Nursing and Allied Health Literature
CROMs	Clinician-Reported Outcomes Measures
MAAT	Mixed Methods Appraisal Tools
NBH	Nursing Bedside Handover
PFPS	Patients for Patient Safety
PREMs	Patient-Reported Experience Measures
PRISMA	Preferred Reporting Items for Systematic reviews and Meta-Analyses
PROMs	Patient-Reported Outcomes measures
SANRA	Scale for the Assessment of Narrative Review Articles
SotA	State-of-the-art
WHO	World Health Organization

## Appendix A

### Appendix A.1

**Table A1:** Search strategy by database.

Search Strategy	
PubMed	((“patient participation”[Title/Abstract] OR “patient involvement”[Title/Abstract] OR “patient empowerment”[Title/Abstract] OR “patient engagement”[Title/Abstract] OR “patient-centered”[Title/Abstract]) OR “person-centered”[Title/Abstract])) AND (“bedside handover”[Title/Abstract] OR “bedside handoff”[Title/Abstract] OR “bedside clinical handover”[Title/Abstract] OR “bedside clinical handoff”[Title/Abstract] OR “bedside shift-to-shift handover”[Title/Abstract] OR “bedside shift-to-shift handoff”[Title/Abstract] OR “bedside shift report”[Title/Abstract] OR “Change-of-shift bedside report”[Title/Abstract] OR “change-of-shift bedside handover”[Title/Abstract] OR “change-of-shift bedside handoff”[Title/Abstract] OR “shift report at bedside”[Title/Abstract] OR “bedside sign-in”[Title/Abstract] OR “bedside sign-out”[Title/Abstract] OR “shift report”[Title/Abstract] OR “shift change”[Title/Abstract] OR “nursing handover”[Title/Abstract] OR “nursing handoff”[Title/Abstract])) AND (nurs*[Title/Abstract])
CINAHL Complete	AB (“patient participation” OR “patient involvement” OR “patient empowerment” OR “patient engagement” OR “patient-centered” OR “person-centered”) AND AB (“bedside handover” OR “bedside handoff” OR “bedside clinical handover” OR “bedside clinical handoff” OR “bedside shift-to-shift handover” OR “bedside shift-to-shift handoff” OR “bedside shift report” OR “Change-of-shift bedside report” OR “change-of-shift bedside handover” OR “change-of-shift bedside handoff” OR “shift report at bedside” OR “bedside sign-in” OR “bedside sign-out” OR “shift report” OR “shift change” OR “nursing handover” OR “nursing handoff”) AND AB nurs*
Scopus	(ABS (“patient participation” OR “patient involvement” OR “patient empowerment” OR “patient engagement” OR “patient-centered” OR “person-centered”) AND ABS (“bedside handover” OR “bed-side handoff” OR “bedside clinical handover” OR “bedside clinical handoff” OR “bedside shift-two-shift handover” OR “bedside shift-two-shift handoff” OR “bedside shift report” OR “change-off-shift bedside report” OR “change-off-shift bedside handover” OR “change-off-shift bedside handoff” OR “shift report rat bedside” OR “bedside sign-ion” OR “bedside sign-out” OR “shift report” OR “shift change” “nursing handover” OR “nursing handoff”) AND ABS (nursing))

### Appendix A.2

**Table A2:** Results from the critical appraisal using the Mixed Methods Appraisal Tool (MMAT).

SN	Author(s) and Year	Q1	Q2	Q3	Q4	Q5	Score
1.	Dumbala et al. [91]	Yes	Yes	Yes	Yes	Yes	10 ^a^
2.	Yang et al. [93]	Yes	Yes	Yes	Yes	Yes	10 ^e^
3.	Ghosh et al. [109]	Yes	Yes	Yes	Yes	Yes	10 ^c^
4.	Abt et al. [78]	Yes	Yes	Yes	Yes	Yes	10 ^e^
5.	Ghosh et al. [120]	Yes	Yes	Yes	Cannot tell’	Yes	9 ^c^
6.	Cruchinho et al. [84]	Yes	Yes	Yes	Yes	Yes	10 ^d^
7.	Casey et al. [99]	Yes	Yes	Yes	Cannot tell’	Cannot tell’	8 ^a^
8.	Chien et al. [101]	Yes	Yes	Yes	Yes	Yes	10 ^a^
9.	Van de Velde et al. [76]	Yes	Yes	Cannot tell’	Cannot tell’	Cannot tell’	7 ^a^
10.	Lantz et al. [83]	Yes	Yes	Cannot tell’	Cannot tell’	Yes	8 ^b^
11.	Yuen et al. [108]	Yes	Yes	Yes	Cannot tell’	Yes	9 ^a^
12.	Paredes-Garza et al. [85]	Yes	Yes	Cannot tell’	Cannot tell’	Yes	8 ^a^
13.	Dahm et al. [104]	Yes	Yes	Yes	Yes	Cannot tell’	9 ^a^
14.	Tobiano et al. [100]	Yes	Yes	Yes	Cannot tell’	Cannot tell’	9 ^d^
15.	Neamti et al. [89]	Yes	Yes	Cannot tell’	Cannot tell’	Yes	8 ^b^
16.	Chien et al. [102]	Yes	Yes	Yes	Yes	Yes	10 ^a^
17.	Street et al. [98]	Yes	Yes	Yes	Yes	Yes	10 ^a^
18.	Abbaszade et al. [90]	Yes	Yes	Yes	Cannot tell’	Yes	9 ^b^
19.	Olasoji et al. [107]	Yes	Yes	Yes	Yes	Yes	10 ^a^
20.	Mullen et al. [103]	Yes	Yes	Yes	Cannot tell’	Yes	9 ^a^
21.	Chong et al. [92]	Yes	Yes	Yes	Cannot tell’	Yes	9 ^d^
22.	Forde et al. [87]	Yes	Yes	Yes	Yes	Yes	10 ^c^
23.	Wiklund et al. [81]	Yes	Yes	Yes	Yes	Yes	10 ^a^
24.	Oxelmark et al. [79]	Yes	Yes	Yes	Yes	Yes	10 ^d^
25.	Malfait et al. [77]	Yes	Yes	Cannot tell’	Cannot tell’	Yes	8 ^b^
26.	Hada et al. [94]	Yes	Yes	Cannot tell’	Cannot tell’	Yes	8 ^a^
27.	Kullberg et al. [82]	Yes	Yes	Yes	Yes	Yes	10 ^d^
28.	Olasoji et al. [106]	Yes	Yes	Yes	Cannot tell’	Yes	9 ^d^
29.	Malfait et al. [74]	Yes	Yes	Cannot tell’	Cannot tell’	Yes	8 ^b^
30.	Benham-Hutchins et al. [115]	Yes	Yes	Cannot tell’	Cannot tell’	Cannot tell’	6 ^a^
31.	Malfait et al. [75]	Yes	Yes	Yes	Cannot tell’	Yes	9 ^b^
32.	Olasoji et al. [105]	Yes	Yes	Yes	Cannot tell’	Yes	9 ^a^
33.	Malfait et al. [73]	Yes	Yes	Yes	Yes	Cannot tell’	9 ^c^
34.	Kullberg et al. [80]	Yes	Yes	Yes	Yes	Yes	10 ^a^
35.	Benham-Hutchins et al. [116]	Yes	Yes	Yes	Cannot tell’	Yes	9 ^a^
36.	Tobiano et al. [95]	Yes	Yes	Cannot tell’	Cannot tell’	Yes	8 ^a^
37.	Scheidenhelm and Reitz [25]	Yes	Yes	Yes	Cannot tell’	Yes	9 ^b^
38.	Khuan and Juni [7]	Yes	Yes	Yes	Cannot tell’	Yes	9 ^a^
39.	Witty et al. [96]	Yes	Yes	Yes	Cannot tell’	Yes	9 ^d^
40.	Bruton et al. [118]	Yes	Yes	Yes	Yes	Yes	10 ^a^
41.	Lupieri et al. [86]	Yes	Yes	Yes	Cannot tell’	Yes	9 ^a^
42.	Eggins and Slade [97]	Yes	Yes	Yes	Cannot tell’	Yes	9 ^a^
43.	Drach-Zahavy and Shilman [88]	Yes	Yes	Yes	Cannot tell’	Yes	9 ^c^
44.	Lu et al. [110]	Yes	Yes	Yes	Cannot tell’	Yes	9 ^a^
45.	Bradley and Mott [111]	Yes	Cannot tell’	Yes	Cannot tell’	Yes	8 ^c^
46.	San-Jecklin and Sherman [117]	Yes	Yes	Cannot tell’	Cannot tell’	Cannot tell’	7 ^b^
47.	Johnson and Cowin [112]	Yes	Yes	Yes	Cannot tell’	Yes	9 ^a^
48.	McMurray et al. [113]	Yes	Yes	Yes	Cannot tell’	Yes	9 ^a^
49.	McMurray et al. [114]	Yes	Yes	Yes	Cannot tell’	Yes	9 ^a^
50	Greaves [119]	Cannot tell’	Yes	Yes	Cannot tell’	Cannot tell’	7 ^a^

(a) [Tool for qualitative studies]—Q1: Is the qualitative approach appropriate to answer the research question? Q2: Are the qualitative data collection methods adequate to address the research question? Q3: Are the findings adequately derived from the data? Q4: Is the interpretation of results sufficiently substantiated by data? Q5: Is there coherence between qualitative data sources, collection, analysis, and interpretation? [68]. (b) [Tool for quantitative non-randomized studies]—Q1: Are the participants representative of the target population? Q2: Are measurements appropriate regarding both the outcome and intervention (or exposure)? Q3: Are there complete outcome data? Q4: Are the confounders accounted for in the design and analysis? Q5: During the study period, is the intervention administered (or exposure occurred) as intended? [68]. (c) [Tool for mixed methods studies]—Q1: Is there an adequate rationale for using a mixed methods design to address the research question? Q2: Are the different components of the study effectively integrated to answer the research question? Q3: Are the outputs of the integration of qualitative and quantitative components adequately interpreted? Q4: Are divergences and inconsistencies between quantitative and qualitative results adequately addressed? Q5: Do the different components of the study adhere to the quality criteria of each tradition of the methods involved? [68]. (d) [Tool for quantitative descriptive studies]—Q1: Is the sampling strategy relevant to address the research question? Q2: Is the sample representative of the target population? Q3: Are the measurements appropriate? Q4: Is the risk of nonresponse bias low? Q5: Is the statistical analysis appropriate to answer the research question? [68]. (e) [Tool for quantitative randomized controlled trials]—Q1: Is randomization appropriately performed? Q2: Are the groups comparable at baseline? Q3: Are there complete outcome data? Are outcome assessors blinded to the intervention provided? Did the participants adhere to the assigned intervention? [68].
