# Supplementary material for: Patient Participation During Nursing Bedside Handover: A State-of-the-Art Review
Source: Nurs Rep. 2025 Dec 10;15(12):438. doi: 10.3390/nursrep15120438 (PMC12736045; doi:10.3390/nursrep15120438)
Supplement: Supplementary file 1 [file nursrep-15-00438-s001.zip › Table S1.pdf]

Scale for the Assessment of Narrative Review Articles — SANRA[1]

Justification of the article's importance for the readership	Score	Location*
0. The importance is not justified<br>1. The importance is alluded to, but not explicitly justified<br>2. The importance is explicitly justified	2	Lines 176-179
**Statement of concrete aims or formulation of question**	**Score**	**Lines**
0. No aims or questions are formulated<br>1. Aims are formulated generally but not concretely or in terms of clear questions<br>2. One or more concrete aims or questions are formulated	2	Lines 170-172, 208-210
**Description of the literature search**	**Score**	**Lines**
0. The search strategy is not presented<br>1. The literature search is described briefly<br>2. The literature search is described in detail, including search terms and inclusion criteria	2	Lines 235-290, 1279
**Referencing**	**Score**	**Lines**
0. Key statements are not supported by references<br>1. The referencing of key statements is inconsistent<br>2. Key statements are supported by references	2	Lines 718-1135
**Scientific reasoning**	**Score**	**Lines**
0. The article's point is not based on appropriate arguments<br>1. Appropriate evidence is introduced selectively<br>2. Appropriate evidence is generally present	2	Lines 40-179
**Appropriate presentation of data**	**Score**	**Lines**
0. Data are presented inadequately<br>1. Data are often not presented in the most appropriate way<br>2. Relevant outcome data are generally presented appropriately	2	Lines 350-710

[1] Adapted in checklist reporting format. **Source:** Baethge, C.; Goldbeck-Wood, S.; Mertens, S. SANRA: A Scale for the Quality Assessment of Narrative Review Articles. *Res. Integr. Peer Rev.* **2019**, *4*, 5, doi:10.1186/s41073-019-0064-8. This work is licensed under CC BY 4.0. To view a copy of this license, visit https://creativecommons.org/licenses/by/4.0/ .

* Examples in the manuscript.
