# Supplementary material for: Patient Participation During Nursing Bedside Handover: A State-of-the-Art Review
Source: Nurs Rep. 2025 Dec 10;15(12):438. doi: 10.3390/nursrep15120438 (PMC12736045; doi:10.3390/nursrep15120438)
Supplement: Supplementary file 1 [file nursrep-15-00438-s001.zip › Table S2.pdf]

Table S2. Summary of included papers and the appraisal quality obtained.

SN	Author(s) and year	Aim(s)	Study context	Study design and participants	Results	Methodological quality
1.	Dumbala et al. [89]	To explore nurses' experiences with clinical handover	Medical-surgical units and outpatient emergency services at Jimma Medical Center in Southwestern Ethiopia, some of which have implemented NBH	Qualitative study using a phenomenological approach. Semi-structured, in-depth interviews with nine nurses and five intentionally selected key informants, along with non-participant observation of nurses' handovers.	–Patients and family members are not intentionally involved in the handover process<br>–NBH is mainly performed when patients experienced recent hemodynamic changes<br>–No detailed personal information is reported during the handover<br>–Some departments do not have an organizational handover policy or a clear description of activities necessary for an effective clinical handover	Strong
2.	Yang et al. [91]	To design and explore the influence of a model combining the ISOBAR communication tool with the case teaching method	Department of Pediatrics, First Affiliated Hospital of Sun Yat-Sen University (China). One group of nurses implemented ISOBAR combined with case teaching, while another used the	Experimental study design with 40 nurses (experimental group) and 44 nurses (control group). Outcomes were assessed two months after standardized training using the Nursing Assessment of Shift Report (NASR) scale, measures of clinical nursing ability,	–The experimental group was significantly better than the control group in terms of a) patient safety assurance; b) patient participation promotion; c) enhancement of nurses' supervision; d) cooperation; and e) responsibility.<br>–The experimental group scored significantly higher (27.75 ± 1.4) in the "patient participation promotion" dimension than the control group (24.07 ± 3.91), validating the effectiveness of this approachthis teaching method in promoting patient participation.	Strong

SN	Author(s) and year	Aim(s)	Study context	Study design and participants	Results	Methodological quality
			traditional lecture method combined with case teaching.	satisfaction with teaching, willingness to continue working in nursing, and incidence of adverse nursing events		
3.	Ghosh et al. [99]	To explore patients' perceptions about their involvement in nursing bedside handovers in acute hospitals	Public and private hospitals, where the NBH policy in the public hospital required handover to be conducted at the bedside with the patient and the multidisciplinary care team whenever possible, while in the private hospital NBH was required to occur at the beginning of the afternoon shift. Additionally, the public hospital	Mixed-method study using a questionnaire with closed and open-ended questions applied to 442 patients, 333 from the private hospital and 109 from the public hospital. Open-ended responses were analyzed using content analysis	– 18% of patients stated they were unaware of the possibility of participating in NBH or were "never" asked to participate, with discussions occurring only between nurses and limited to name and date of birth exchanges<br>–9% reported that handovers occurred outside the room in the hallway, excluding them from the conversation<br>–14% mentioned NBH occurred at times when involvement was not feasible, such as while they were asleep or when family members were not present<br>–To improve engagement, 19% suggested raising awareness and inviting patients and families, and 22% proposed standardizing handover with patient and family involvement<br>–52% indicated that involvement helped them stay informed about their care, understand treatment progress, and clarify concerns, while 17% noted it	Strong

SN	Author(s) and year	Aim(s)	Study context	Study design and participants	Results	Methodological quality
			used the ISOBAR protocol, and the private hospital used the SHARED protocol		reduced anxiety and increased trust in care	
4.	Abt et al. [76]	To evaluate the feasibility, acceptability and preliminary effectiveness of I-PASS-structured (Identification, Patient, Action, Situation, Synthesis) bedside nursing handovers on handover global quality and patients' trust in nurses	Surgical and medical wards of a Swiss hospital, where nurses received NBH training including simulation sessions, working on their beliefs and helping them to reflect on the partnerships established with hospitalized patients.	Type-1 effectiveness–implementation hybrid study. Semi-structured interviews with nurses and patients, and the application of some instruments, such as a questionnaire to assess patient perception, the Treatment Acceptability and Preferences (TAP) measure, the Trust in Nurses Scale (TNS), and the Manser Rating Tool for Handoff Quality (RTHQ)	- Combining structured nurse-to-nurse information transfer with patient involvement improved handover quality compared to traditional handovers.<br>- Reported obstacles: time constraints, confidentiality concerns, and patient interruptions<br>- Reported facilitators: coaching, advance organization, standardized handovers, and training	Strong

SN	Author(s) and year	Aim(s)	Study context	Study design and participants	Results	Methodological quality
5.	Ghosh et al. [118]	To explore the perceptions of patients and families about their involvement in bedside handover	All wards and units (medical, surgical, critical care, and mental health) in two Australian hospitals (one public and one private) that used the iSoBAR and SHARED protocols	Mixed-method study including a cross-sectional survey with open and closed questions followed by semi-structured interviews with a purposive and convenience sampling of 24 patients and family members	- Patients stated that: 1) Improved communication with nurses increased the quality of care received, resulting in more personalized and responsive treatment; 2) Involvement in their care made them feel more informed and empowered; 3) Nurses' compassion, kindness, and attentiveness significantly reduced hospitalization anxiety and promoted a sense of safety;4) Participation in NBH improved understanding of their health condition and empowered them to contribute to their care; 5) Knowing about the possibility of participating in NBH would have boosted their confidence to ask questions; 6) They would have liked their family to be present during NBH when they were unable to participate<br>- Family members reported that their participation in the handover helped them identify and address issues needing attention, such as discharge planning, especially for elderly patients, those with dementia, or those not speaking English-Barriers identified: 1) organizational (a lack of information to patients about the possibility of participating and NBH timing not aligned with visiting hours); 2) nurse-related (handovers done in	Strong

SN	Author(s) and year	Aim(s)	Study context	Study design and participants	Results	Methodological quality
					hallways or behind curtains, lack of invitations for participation); 3) patient-related (fear of asking questions and health condition limitations)	
6.	Cruchin-ho et al. [82]	To perform a cross-cultural validation of the Bedside Handover Attitudes and Behavior Questionnaire for the Portuguese population and to analyze its psychometric properties	The study was conducted in a hospital institution that uses NBH	Quantitative, cross-sectional, and descriptive study involving a sample of 241 nurses from various inpatient contexts	– The validation of the assessment instrument revealed a factor structure consisting of 18 items distributed across four dimensions, leading the researchers to propose a new Conceptual Model for Patient Participation in Nursing Bedside Handover, composed of the following four dimensions: 1) Direct Engagement; 2) Personal Interaction; 3) Information Sharing, and 4) Individualized Approach	Strong
7.	Casey et al. [108]	To explore patient and nurse perceptions of a prototype App designed to support nurse-patient communication	Patients from a medical/oncology ward in Australia used the App to ask questions and communicate their healthcare preferences, which nurses	Qualitative study with an exploratory and descriptive approach using a combination of questionnaires, observations, and interviews involving 22 nurse-patient	- Despite persistent barriers to patient involvement during NBH, many patients (n=11) and nurses (n=8) reported that the app positively influenced patient engagement during NBH	Strong

SN	Author(s) and year	Aim(s)	Study context	Study design and participants	Results	Methodological quality
		and patient engagement	addressed during their shifts	dyads recruited by convenience		
8.	Chien et al. [110]	To improve the effectiveness of NBH through a qualitative, tailored communication intervention	Implementation of an intervention combining training and context-specific practical change recommendations co-designed with seven wards from three Australian hospitals	Multi-site before-and-after study with an ethnographic approach combined with discourse analysis of interactions during NBH	– After implementing the intervention, nurses routinely conducted the handover at the bedside, started and ended the handover more punctually, valued NBH as a patient-centered approach, and began using the CARE protocol to actively involve patients in the NBH	Strong
9.	Van de Velde et al. [74]	To investigate "Experience-based co-design" as a suitable method for formulating recommendations regarding the goals, structure, content, and familiarization process of NBH in mental health care	Mental health care unit of a general hospital in Belgium aiming to implement NBH recommendations in mental health settings	Qualitative study design combining semi-structured interviews and two focus groups involving patients, nurses, and other healthcare professionals	–Using the "Experience-based co-design" method, patients and peer support workers expressed that NBH should allow for more dialogue with nurses, including asking questions, requesting clarification, setting clear expectations, and participating in joint decision-making with the nursing team<br>–Healthcare providers stated that the purpose of NBH was to enhance patients' sense of safety and involvement	Moderate

SN	Author(s) and year	Aim(s)	Study context	Study design and participants	Results	Methodological quality
10.	Lantz et al. [81]	To evaluate patient participation in relation to Person-Centered Handover	The study was conducted in nine units of a university hospital in Sweden that adopted a policy to involve patients during NBH and implemented a training program to support the Person-Centered Handover. Nurse managers were part of the implementation team	Pretest-post-test study design without a comparison group, using the Patient Participation Tool to assess preference and experience of participation with a sample of 228 patients (pretest) and 253 patients (post-test)	– Only 120 patients in the post-test group received Person-Centered Handover; those who received it showed higher levels of participation compared to those who did not, particularly in: 1) sharing one's symptoms with staff; 2) reciprocal communication; 3) being told what was done; and 4) taking part in planning	Strong
11.	Yuen et al. [98]	To assess the feasibility and effectiveness of a digital app developed to improve patient-nurse communication during NBH	Study conducted in Australia with a co-designed digital app enabling patients to: 1) understand the handover process; 2) identify key	Qualitative study analysing video recordings of 12 simulated handovers conducted in a simulation lab replicating an acute care ward; involved six experienced	–During NBH, all patients were given the opportunity to ask questions. Although most of the questions had been submitted earlier via the app, 58% of patients asked additional questions during the NBH<br>–Nurses showed high levels of communication behaviours including: 1) greeting the patient warmly; 2)	Strong

SN	Author(s) and year	Aim(s)	Study context	Study design and participants	Results	Methodological quality
			information; 3) ask questions to be answered by their nurse; and 4) express any concerns	nurses and 11 patient actors/volunteers, each with the app installed on an iPad	demonstrating good nonverbal behaviour; 3) allowing time for the patient to absorb information; 4) giving clear explanations; 5) involving the patient in decisions; and 6) exploring the acceptability of the care plan. However, they showed low levels of encouraging emotional expression and accepting patient feelings<br>– Authors concluded that the app has the potential to educate and empower patients/carers to be active partners in communication with nurses during NBH	
12.	Paredes-Garza et al. [83]	To understand the perception of nursing professionals about the influence of the infrastructure of critical care units during NBH	The study was conducted in a polyvalent critical care unit in Spain that had not implemented an organized NBH	Qualitative study using a Grounded Theory approach through semi-structured interviews	Greater patient participation in NBH was described as a clarifying element of their health status, which could influence nurses' actions and engage patients in their recovery process	Strong
13.	Dahm et al. [94]	To identify how patient empowerment and	This study was conducted in three wards of two metropolitan	Qualitative study with a multiple case study approach, combining	– In most observed NBHs, nurses ending their shift did not greet patients, referred to them using impersonal third-person pronouns or terms of endearment, used	Strong

SN	Author(s) and year	Aim(s)	Study context	Study design and participants	Results	Methodological quality
		participation can be shaped by observed communication behaviours during NBH	hospitals in Australia that had implemented NBH	ethnographic observation (observing, note-taking, and digitally recording handover interactions) of 38 NBHs	technical jargon, ignored contributions from patients or accompanying persons, and used task-oriented power interruptions to end patients' narratives or to shorten incoming nurses' engagement. In contrast, incoming nurses actively engaged with patients through introductions, emphatic use of first names, gathering patients' opinions, and actively participating in their contributions during NBH	
14.	Tobiano et al. [109]	To systematically develop and psychometrically evaluate a self-report questionnaire to measure patients' perceptions of participation during NBH	The study was conducted in a metropolitan tertiary hospital in Australia	Quantitative, cross-sectional, and descriptive study including 326 patients from five services: 1) gastrointestinal medicine and surgery; 2) cardiology medicine; 3) neurology medicine; 4) vascular medicine and surgery and 5) head and neck, urology, and gynecology surgery	–The developed instrument has 24 items distributed across three reliable factors: 1) Conditions for patient participation in bedside handover (α = 0.96); 2) Level of patient participation in the handover, especially related to communication (α = 0.87); and 3) Evaluation of patient participation in bedside handover (α = 0.96)<br>–The difference in scores for these factors when handover is conducted at the bedside versus away from patients highlighted the importance of nurses' facilitation behaviours as a critical success factor for active patient participation in NBH	Strong

SN	Author(s) and year	Aim(s)	Study context	Study design and participants	Results	Methodological quality
15.	Neamati et al. [87]	To investigate the effect of the I PASS the BATON protocol (which includes patient involvement in the handover) on the quality of nursing handover	This study was conducted in two public hospitals in Iran. In one hospital, a training-based intervention on the use and effects of the I PASS the BATON protocol was implemented to reduce errors and improve the quality of nursing care. In the other hospital, nurses continued using the SBAR protocol	Quantitative, quasi-experimental design that evaluated handover quality based on self-report using the Handoff Evaluation Scale and through non-participant observation before the intervention and two months later, in a sample of 34 nurses (intervention group) and 31 nurses (control group)	–Statistically significant differences were found between the two groups after the intervention, particularly in the quality of information, interaction and support, efficiency, and patient involvement ($p < 0.05$), which were attributed to the use of the I PASS the BATON protocol	Strong
16.	Chien et al. [111]	Improving the patient-centredness of nursing handover by addressing communication challenges and	The study was conducted in a rehabilitation ward of an Australian metropolitan teaching hospital, which	Qualitative study using a qualitative approach to analyse NBHs, combined with interviews (n = 27 before the intervention and n = 6 after the	–Before the intervention, the handover was inappropriately located (in the hallway), there was a lack of patient involvement, and the information structure was absent linked to systemic and cultural factors in the ward's organizational and cultural context<br>–After the intervention, the NBH was	Strong

SN	Author(s) and year	Aim(s)	Study context	Study design and participants	Results	Methodological quality
		the organizational and cultural practices that shape handover	implemented and evaluated a tailored intervention that simultaneously addressed communication challenges and the range of situated practices that enabled and constrained patient-centred communication during handover, supervised by the nurse manager	intervention), two focus groups (n = 7), and observation of 16 NBHs and 2 huddles	conducted at the bedside, nurses began to more actively involve and interact with patients during the handover, and they used the CARE and ISBAR protocols to structure their interactions	
17.	Street et al. [107]	To explore the frequency and nature of patient participation in nursing handover and the strategies perceived by patients and nurses to increase patient	The study involved the observation of 117 NBHs and classified patient participation into: 1) an active participant (asks questions, makes statements about their condition);	Multi-site prospective study with a mixed-methods approach combining observation and interviews with 33 patients and 20 nurses recruited from 10 wards across six Australian	–76.9% (n = 90) of the NBHs were conducted in the presence of the patient, of which only 33.3% (n = 30) had patients actively participating, and 46.7% (n = 42) had patients as passive participants<br>–Additionally, patients and nurses recognized the value of patient participation in NBH as a platform for information exchange that facilitates understanding and enhances patient safety	Strong

SN	Author(s) and year	Aim(s)	Study context	Study design and participants	Results	Methodological quality
		involvement in NBH	2) a passive participant (nods or makes short superficial comments); or 3) no participation (no form of patient communication during handover)	hospitals	– Nurse behaviours associated with active participation during handover included providing explanations during the handover ($p = .005$), making eye contact with the patient ($p = .001$), asking direct questions ($p < .001$) or for clarification ($p < .001$), and giving patients or visitors the opportunity to ask questions ($p < .001$)	
18.	Abbas-zade et al. [88]	To evaluate the impact of NBH on nursing care quality using the Situation, Background, Assessment, Recommendati-on (SBAR) technique.	Coronary care units of two public hospitals in Bojnurd (Iran).	Quasi-experimental study involving 14 nurses (trained over five one-hour sessions) and 36 patients, who responded to the Persian version of the Quality Patient Care Scale (QUALPACS) to assess the quality of patient care.	– In both hospital there was substantial increases in the mean score of UALPACS dimensions, namely psychosocial ($P < .001$ and $P <.001$), physical ($P < .001$ and $P = .014$), and communication ($P < .001$ and $P < .001$) after the SBAR implementation.<br>– In the communication dimension, patients and families reported better responses to their questions and needs<br>– Researchers concluded that SBAR technique can affect nurses' performance by facilitating information exchange, reducing the possibility of physical loss of patient information during handover, decreasing the communication load, and reducing patients' concern and anxiety about care.	Strong

SN	Author(s) and year	Aim(s)	Study context	Study design and participants	Results	Methodological quality
19.	Olasoji et al. [97]	To explore the views of patients with a mental illness about their experiences of being involved in nursing handover on an acute mental health inpatient unit following the implementation of a change in nursing handover that involved patients	An adult inpatient mental health unit in a large metropolitan hospital in Australia that was implementing NBH, both in patient rooms and interview rooms, involving patients and their families, preceded by a 10-minute meeting with all nurses and the nurse manager	Qualitative descriptive study design using semi-structured interviews with a sample of 10 patients with various diagnoses, genders, and ages who had been hospitalized for at least five days	– Participants reported that knowing who would be taking care of them in each nursing shift was a benefit of NBH<br>– They also highlighted that NBH gave them the opportunity to be actively involved in the exchange of information about their care between nurses, feeling that their voice was heard through requests for clarification and contributions to their own care planning<br>– Most participants expressed a preference for NBH to take place in private spaces rather than common areas	Strong
20.	Mullen et al. [112]	To explore the experiences and perceptions of nursing staff and nursing managers about the broader concept of	The study was conducted in three mental health units of an Australian hospital that were unsuccessful in implementing NBH with patient	Qualitative descriptive study using six focus groups with 20 nurses and 11 individual interviews with nurse managers	– Both nurses and nurse managers expressed that NBH was not being implemented in all units or was occurring inconsistently<br>– Nurses also considered that patient involvement could constitute a breach of confidentiality since patients in the same ward could overhear the information discussed	Strong

SN	Author(s) and year	Aim(s)	Study context	Study design and participants	Results	Methodological quality
		involving consumers in nursing handover	involvement		– Nurses agreed that the information shared in NBH should be filtered<br>– Nurse managers attributed the nurses' reluctance to involve patients to a lack of confidence or skills in determining what information to share and how to adjust the language<br>– It was also noted that implementing NBH required a change management approach involving nurse participation and training	
21.	Chong et al. [90]	To examine nurses' perceptions of intershift handover, especially the quality of information, efficiency, interaction and support, as well as patient involvement	Tertiary public hospital in Malaysia using bedside, written (manual, not electronic), and face-to-face verbal handover with a standardized and structured approach within each ward	Quantitative, cross-sectional, and descriptive study using the Handover Evaluation Scale with a sample of 414 nurses from medical, surgical, orthopaedic, obstetrics and gynaecology, and paediatric wards. Included one open-ended question to identify strengths and limitations of the handover practice	–Participants reported lower perceptions of patient involvement during NBH compared to interaction, support, and quality of information<br>–Patient involvement during NBH was mentioned by nurses both as a strength (for understanding patient needs) and as a limitation (as it could prolong the handover duration)	Strong

SN	Author(s) and year	Aim(s)	Study context	Study design and participants	Results	Methodological quality
22.	Forde et al. [85]	To describe the structures, processes, and content of NBH in the acute care context	Acute care hospital in Ireland with one year of NBH implementation	Convergent parallel mixed-methods design combining observation and audio recording of 30 handover episodes	– Nurses going off shift appeared to influence the level of patient participation, as they focused on conveying patient information, assessments, and care plans<br>– Nurses coming on shift were observed receiving and processing the information with little verification or clarification	Strong
23.	Wiklund et al. [79]	To explore patients' experiences of bedside handover during childbirth	Conducted in a maternity clinic in Sweden	Qualitative descriptive study using semi-structured interviews with 12 couples, parents of healthy children, who experienced bedside handover during childbirth	– Parents reported that although they were not always verbally active during the handover, they felt involved in the process and paid close attention to non-verbal communication from nurses, such as whether they were acknowledged or ignored<br>– They also stated that: 1) Knowing which nurse was starting the shift reduced their anxiety; 2) Participating in the handover gave them a sense of safety and security in relation to their care; 3) The use of medical jargon caused concern; and 4) The partner could actively participate in the handover when the mother was unable to due to pain	Strong
24.	Oxel-mark et al. [77]	To describe differences and similarities in	Conducted in departments of two Swedish	Discrete choice experiment including 218 patients and 101	– Patients perceived their level of participation in handover as highly important, with the ability to speak up	Strong

SN	Author(s) and year	Aim(s)	Study context	Study design and participants	Results	Methodological quality
		preferences between patients and nurses regarding the implementation of NBH	university hospitals that had not yet implemented NBH	nurses	and to hear what is said being the most important feature<br>– Nurses also attributed high importance to patient participation, particularly the importance of inviting patients to participate and enabling them to be listened to and speak	
25.	Malfait et al. [75]	To discuss and explore privacy issues during patient transfers in bed from different perspectives	Twelve wards (5 surgical, 4 medical rehabilitation, and 3 geriatric) across 7 different hospitals in the Flemish region of Belgium.	Controlled, multicentred, and longitudinal study on feasibility, appropriateness, meaningfulness, and effectiveness of the NBH. 48 patient interviews, 106 nurse interviews, and 638 non-participant observations of patient transfers	– Patients considered nursing care information to be less sensitive and trusted nurses' judgment on what could be disclosed.<br>– Patients reported that they often shared information spontaneously with the patient next to them, since they had no personal ties with them.<br>– About 60% of nurses expressed reluctance to perform NBH due to privacy concerns	Strong
26.	Hada et al. [92]	To systema-tically assess the barriers and facilitators to evidence-based handover and identify	Conducted in four medical wards of a tertiary hospital in Australia that use NBH	Qualitative study design with five focus groups involving a sample of 49 nurses	– "Patient involvement" emerged as a sub-theme within the "process" theme, with two categories: 1) inviting the patient to confirm/clarify and 2) inviting the patient to ask questions<br>– Participants reported that patient involvement is very superficial and	Strong

SN	Author(s) and year	Aim(s)	Study context	Study design and participants	Results	Methodological quality
		potential adopters and attributes of evidence-based nursing handover for practical implementation			influenced by nurses' concerns about confidentiality, patients' reluctance to participate in discussions—especially when many nurses are present during handover—and time constraints<br>– Suggested facilitators for increasing confidence in handling confidential information included targeted education and clear guidelines<br>– Nurses agreed that patient involvement in handover can increase their participation in care planning	
27.	Kullberg et al. [80]	To investigate patients' satisfaction with care in an oncological inpatient setting, and to describe patients' perceptions of individualized care	Conducted in two inpatient oncology wards at a Swedish hospital using Person-Centered Handover for two years, where patients are encouraged to share their daily care experiences, ask questions, and speak up	Longitudinal quantitative study using two-timepoint measures with a subscale from the "EORTC INPATSAT-32" questionnaire on information exchange and the "Individualized Care Scale", in a sample of 90 patients	– A statistically significant improvement was found in patient satisfaction with information exchange two years after the introduction of Person-Centered Handover, which authors attributed to patients being able to see and hear what was being communicated, though it did not necessarily indicate active participation<br>– Patients also reported high scores for the degree to which individualized care was supported and perceived	Strong

SN	Author(s) and year	Aim(s)	Study context	Study design and participants	Results	Methodological quality
28.	Olasoji et al. [96]	To identify nurses' attitudes toward patient involvement during nursing handover	Conducted in an adult mental health unit at a metropolitan hospital in Australia that had not yet implemented NBH, where a training program for nurses was delivered using five case-based scenarios illustrating basic principles of patient involvement in nursing handover via short video vignettes	Quantitative exploratory and descriptive study with a pre-post design in a single group, using a 31-item questionnaire before the intervention and a 35-item questionnaire after, to assess nurses' (n=36 pre, n=27 post) opinions on proposed changes to the handover process	– The effectiveness of the intervention was inversely associated with participants' age and positively associated with the appropriateness of discussions with patients during handovers and the opportunity to strengthen the therapeutic relationship with patients<br>– It was also positively associated with years of experience in mental health nursing and negatively associated with negative attitudes toward content in nursing handover.	Strong
29.	Malfait et al. [72]	To investigate the effects of bedside handover on nurses and patients	The study involved a total of 13 wards from five Belgian hospitals (medical rehabilitation, geriatric, and	Non-randomized, longitudinal, multicenter quantitative study including 524 patients and 91 nurses in the	– Although the intervention included a six-hour educational program, no statistically significant differences were found in patient-reported outcomes over time between the group without NBH and the group with NBH in terms of patient empowerment	Strong

SN	Author(s) and year	Aim(s)	Study context	Study design and participants	Results	Methodological quality
			surgical/medical wards), including six wards with traditional handovers and seven wards that implemented NBH.	intervention group and 245 patients and 74 nurses in the control group	–Conversely, nurses reported statistically significant differences regarding individualized care and the level of patient participation in care	
30.	Benham-Hutchins et al. [113]	To identify the health information that Spanish-speaking Hispanic patients want and need during hospitalization and to explore their opinions on including these needs in the NBH and interdisciplinary rounds held in their hospital room	The study involved recently hospitalized patients in wards that had implemented NBH with patient involvement in communication	Qualitative study using two focus group interviews with a sample of eight patients with cancer and diabetes as primary diagnoses, recently hospitalized. The effects of NBH were measured on patients (patient empowerment, quality of care, and individualized care) and nurses (job satisfaction, turnover intention, coordination of care process,	– Most participants reported that despite being able to participate in the handover communication, they did not have the opportunity to be involved in discussions about their treatment plan to learn how to self-manage their condition<br>– They also reported that in some circumstances, the NBH included only the introduction of the incoming nurse without discussing the treatment plan and that sometimes no introductions occurred before the shift change	Moderate

SN	Author(s) and year	Aim(s)	Study context	Study design and participants	Results	Methodological quality
				communication with patients and family, work interruptions, individualized care, and level of patient participation)		
31.	Malfait et al. [73]	To determine the compliance rates of a structured bedside handover protocol in which patient participation is an essential element, following the ISBARR protocol	The study involved the development of a structured bedside handover protocol co-designed by patients and nurses, transformed into a checklist, and an educational program relating to patient participation, NBH, and the ISBARR protocol	Multicenter observational study using the developed checklist in 638 observations across 12 wards of different types in seven hospitals (five surgical wards, four wards for medical rehabilitation, and three geriatric wards)	– The item "asking the patient whether (s)he has anything to add or has questions" was the fourth most omitted item in nurse practice during NBH and had a compliance rate of 34.4% across all wards<br>– In nearly 30% of observed cases, nurses unilaterally decided not to conduct the bedside handover, and in about one-third of the cases where it was performed, nurses did not actively involve the patients	Strong
32.	Olasoji et al. [95]	To explore the views of consumers with a mental	: This study was conducted before the implement-tation of NBH to	Qualitative study with an exploratory and descriptive approach through	– Participants expressed that they have a role to play in nursing handovers since it focuses on their care, and that their involvement can have positive benefits	Strong

SN	Author(s) and year	Aim(s)	Study context	Study design and participants	Results	Methodological quality
		illness, who have not had prior involvement in nursing handover, about their need to be involved in nursing handover within an acute mental health inpatient unit	use that information in designing a nursing handover system in a mental health unit of a large metropolitan hospital in Australia	semi-structured interviews with a sample of 11 patients with various diagnoses, genders, and ages, and no experience in the handover process with nurses	– They stated that involvement in NBH would help clarify incorrect inferences affecting their care, allow them to voice concerns, understand their care plan for the shift, and establish a timeframe for aspects of their care within the unit<br>– Additionally, participants said they would not want to be involved in the handover if the location compromised their privacy and that certain aspects, such as mental status, mood, or difficult conversations, should be discussed privately between nurses<br>– Regarding family involvement, participants affirmed that the patient should be the one to accept and allow family participation in the handover	
33.	Malfait et al. [71]	To explore the effects of NBH on handover duration by comparing wards before and after its implementation	This study was conducted in 12 nursing wards of seven hospitals in Belgium that implemented a tailored intervention co-designed by nurses and patients in each	Mixed-methods study with a before-implementation approach, combining interviews with nurses and nurse managers (n=105), unstructured observations (n=40), semi-structured observations (n=50),	– In wards that transitioned to decentralized handover using the ISBARR protocol, or that had already decentralized handover and reintroduced ISBARR, a reduction in overall handover duration and an increase in time per patient were observed<br>– Conversely, in wards that already had decentralized handover and ISBARR in place, there was an increase in time per patient and total handover duration. The	Strong

SN	Author(s) and year	Aim(s)	Study context	Study design and participants	Results	Methodological quality
			ward. This included NBH and a specific action by nurses (asking patients whether the information was clear and if they had any questions or anything to add)	and a semi-structured questionnaire	authors interpreted this as indicating that patient participation in handover may increase its duration	
34.	Kullberg et al. [78]	To describe registered nurses' perceptions of person-centered handover implemented in an inpatient oncology context to promote active patient participation	Two oncology inpatient wards in a Swedish hospital with person-centered handover implemented for about three years	Qualitative study using semi-structured interviews with a sample of 11 nurses	– Some participants described person-centered handover as time-consuming, which led to avoiding active patient involvement out of fear of prolonging the process<br>– Others noted that: 1) nurses standing around the patient's bed; 2) conversations ignoring the patient's presence; and 3) lack of awareness that patients are expected to take an active role in handover, could inhibit participation<br>– It was also stated that person-centered handover enables patients to engage in a structured review of patient safety aspects	Strong

SN	Author(s) and year	Aim(s)	Study context	Study design and participants	Results	Methodological quality
35.	Benham-Hutchins et al. [114]	To describe patients' perceptions of chronic disease self-management during hospitalization and their level of participation in shift change bedside handovers	This study related patient participation during NBH as a strategy to promote patient self-care, particularly self-management of chronic conditions	Qualitative study using an online questionnaire (Patient Activation Measure) with closed and open-ended questions, including 34 patients recruited via social media from online patient support groups using convenience and snowball sampling	– Most participants had high activation levels, indicating strong involvement in chronic disease self-management.<br>– Of the 34 participants, 10 reported that handovers were conducted in patient rooms; some were consistently invited to participate, all listened to the exchange between nurses, some asked and answered questions, and others made corrections<br>– Inhibiting behaviours identified: 1) speaking in low tones as if the patient wasn't present; 2) lack of eye contact; 3) use of technical jargon and acronyms; 4) presence of too many people in the room; 5) treating the patient as a number rather than a person; and 6) lack of time or seeming rushed<br>– Facilitators identified: 1) inviting patients to participate; 2) recognizing the value of their input; 3) speaking to patients instead of about them; and 4) encouraging patients to ask questions	Strong
36.	Tobiano et al. [93]	To explore and understand nurses' perceived barriers to	This study was conducted with a sample of nurses working in acute medical wards at	Qualitative study using a survey and content analysis	– Some nurses expressed discomfort involving patients in the handover due to the presence of other patients and their families, preferring to share sensitive information away from the room or in	Strong

SN	Author(s) and year	Aim(s)	Study context	Study design and participants	Results	Methodological quality
		implementing NBH	a private hospital in Australia where NBH had been implemented		private settings<br>– They also felt that patient and family participation could disrupt handover efficiency by introducing unrelated questions, and that patients' conditions might prevent active participation<br>– Additionally, nurses noted that the presence of too many nurses during handover could inhibit patient participation.	
37.	Sheiden-helm and Reitz [75]	To increase nurses' compliance with bedside reporting and improve patient satisfaction scores	This study was conducted in a community hospital that implemented bedside reporting through a training module addressing the standardized process, staff barriers, and supporting scientific evidence. The implementation process was monitored and	Quasi-experimental study that used random observation before (n=132 nurses) and after implementation (n=202 nurses) and a survey with questions addressing patient satisfaction (n=197+93 before implementation and n=190+99 after) to compare the bedside report in a medical-surgical unit (low bedside report compliance) and an	– Compliance with bedside report before the intervention was 12% in the medical-surgical unit and 55% in the obstetric unit. After the intervention, compliance increased to 85% and 84% one month later, and to 84% and 90.6% four months later, respectively<br>– The authors attributed the increase in nurse compliance to the change management strategy and the use of a standardized strategy through the SBAR protocol	Strong

SN	Author(s) and year	Aim(s)	Study context	Study design and participants	Results	Methodological quality
			supported using the Bedside Handoff Competence Checklist, which includes items related to patient involvement in handover communication	obstetric unit (high compliance), before and after the intervention		
38.	Khuan and Juni [7]	To explore nurses' opinions on the feasibility of involving patients during the NBH	The study was conducted in three inpatient units (medical, surgical, and orthopaedic) of a public hospital in Malaysia that implemented NBH with patient involvement	Qualitative study using five focus group discussions with 5 nurses each, totalling 20 nurses	– A superficial involvement of patients during NBH was reported, linked to a lack of knowledge and inexperience in guiding nurse-patient interactions and in using therapeutic communication skills, as well as a task-oriented mindset among nurses<br>– Participants expressed: 1) nurses prioritized physical assessment and comfort over psychosocial content; 2) involving patients during NBH was impractical due to time constraints and the need to meet the needs of patients with different acuity levels; 3) patients have the right to refuse participation in handover communication; and 4) disclosure of sensitive information could compromise patient dignity and self-	Strong

SN	Author(s) and year	Aim(s)	Study context	Study design and participants	Results	Methodological quality
					esteem if shared during NBH without their consent	
39.	Witty et al. [104]	To describe and compare the preferences of nurses and patients regarding the implementation of NBH	This study was conducted in two medical wards of two metropolitan hospitals in Australia to support the definition of recommendations for improving NBH implementation	Quantitative study based on a discrete choice experiment survey to measure the preferences of nurses (n=205) and patients (n=486) regarding three alternative NBH scenarios	– Both patients and nurses expressed a strong preference for conducting the nursing handover at the patient's bedside rather than elsewhere and similarly assigned high importance to inviting the patient to participate in the handover<br>– For patients, one of the most important handover features was having a family member or friend present, second only to the importance of being asked, having the opportunity to speak, and being able to hear what was being said<br>– In comparison, nurses placed more importance on discussing sensitive information away from patients and less on having family or friends present<br>– Additionally, both nurses and patients preferred a handover involving only the outgoing and incoming nurse rather than the entire nursing team	Strong
40.	Bruton et al. [116]	To understand the purpose, impact, and experience of nurse-to-nurse	The study was conducted in two acute wards of a UK hospital, one medical and the	Qualitative study combining non-participant interviews to explore experiences of	– It was found that the style used by nurses during NBH influenced the level of patient involvement. Some nurses spoke in low voices, looking at the handover sheet, while others positioned themselves	Strong

SN	Author(s) and year	Aim(s)	Study context	Study design and participants	Results	Methodological quality
		handover from both patient and staff perspectives, and the perceived differences between nurse handover and medical ward rounds	other surgical, characterized by high patient turnover predominantly from the emergency department	communication during NBH, its purpose, and nurses' and patients' views on the patient role in NBH, with unstructured observation and field notes of 12 office/station handovers and 12 NBHs	at the bedside speaking at a normal tone and sometimes asked questions and addressed expressed concerns<br>– Patients' opinions and experiences on their involvement in handover varied: 1) some felt involved; 2) others wanted more involvement; and 3) some preferred to passively observe the handover without participating<br>– Nurses noted the advantages of NBH as providing patients the opportunity to correct incorrect information and to ask questions	
41.	Lupieri et al. [84]	To describe the experiences of patients who underwent NBH in the postoperative period of cardiothoracic surgeries	The study was conducted in a cardiothoracic intensive care unit in Italy that had been using NBH for 14 years	Descriptive qualitative study using semi-structured interviews with 14 patients	– Patients reported that: 1) they would like to be more involved in the process, as most of the time nurses spoke among themselves, forgetting that the patients were listening; 2) they wanted more information about their functional recovery; 3) the professional language used by nurses was not always easy to understand; 4) lack of privacy was a real issue, though not significantly concerning to patients; 5) they experienced a sense of safety and protection during NBH, feeling reassured by the completeness and accuracy of the information shared;; and 6) they felt the	Strong

SN	Author(s) and year	Aim(s)	Study context	Study design and participants	Results	Methodological quality
					need to express their feelings and concerns and to be reassured when the clinical course was not positive	
42.	Eggins, and Slade [106]	To assess how effectively nurses conduct NBH	Conducted in a unit of a tertiary public hospital in Australia with high patient turnover, which had recently transitioned from traditional handover to NBH	Qualitative study using discourse analysis, combining observations of NBH with 57 patients and 16 nurses, field notes, interviews, and audio recordings of the NBH	– It was observed that: 1) in 14 out of 57 cases, nurses conducted the handover in the corridor due to infection control concerns, presence of family, or because patients were asleep; 2) only 14 patients were directly invited to participate in the handover; 3) 21 patients were not invited, yet eight of them contributed nonetheless; 4) the remaining patients were not present in the room<br>– Additionally, the authors identified four handover styles: 1) excluding the patient from the NBH; 2) including the patient; 3) viewing the patient as a passive element; and 4) viewing the patient as an active element	Strong
43.	Drach-Zahavy and Shilman [86]	To describe the quality and scope of patient participation in the handover process in relation to their personal	Conducted in five surgical wards, conceptualizing patient participation in NBH as a bidirectional process	Mixed-method cross-sectional study combining structured observation of 100 NBH (audio-recorded and transcribed) with	– Patient personality traits (neuroticism, extraversion, conscientiousness) were negatively associated with nurses' initiative to involve patients in NBH. Personality traits (neuroticism and agreeableness) were positively associated with patients' initiative, while openness to experience was negatively associated	Strong

SN	Author(s) and year	Aim(s)	Study context	Study design and participants	Results	Methodological quality
		attributes and the characteristics of the handover encounters	comprising the initiative of the patient and the initiative of the nurse.	content analysis of the communication during handovers and validated questionnaires	– Additionally, the presence of the nurse manager and companions was positively associated with patients' initiative in NBH, while ward workload was negatively associated<br>– The presence of family members was also positively associated with nurses' initiative to engage patients, while heavy ward workload was negatively associated	
44.	Lu et al. [100]	To explore patients' perceptions of NBH	Three clinical wards (acute medical, acute surgical, and maternity) in an Australian health organization where NBH had been implemented for12 months	Qualitative study with a phenomenological approach, interviewing 10 patients from each the ward (n = 30)	– Patients valued NBH as a means of receiving and contributing important information for their treatment and care<br>– Patients expressed concern about technical jargon, which hindered comprehensionWhen patients were asked whether NBH compromised their privacy and confidentiality, most patients felt comfortable discussing their condition during NBH, although some noted that sensitive issues (e.g., STDs, sexuality, religion, mental illness) should not be discussed	Strong
45.	Bradley and Mott [101]	To empirically examine the process and outcomes of	Three acute hospital wards in rural South Australia that	Mixed method study, pretest post-test evaluative approach involving	– Patients appreciated knowing their assigned nurses, being involved in their own care, being informed by nurses about planned care, answering nurses'	Strong

SN	Author(s) and year	Aim(s)	Study context	Study design and participants	Results	Methodological quality
		NBH implementation	implemented NBH	quantitative (quasi-experimental) and qualitative (ethnographic) elements, which included 9 patients and 48 nurses, using ethnographic interviews to understand patients' perceptions and ethnographic interviews and a questionnaire to understand nurses' perceptions	questions, and being able to ask questions<br>– Nurses identified patient involvement and patient-centered content as key positives<br>– Nurses also reported increased job satisfaction and a greater sense of caring.	
46.	Sand-Jecklin and Sherman [115]	To compare patients and nurses opinions, medication error rates, and patients' falls before and after the NBH implementation	Multiple medical-surgical units (neurology/neurosurgery, orthopedics/plastics, trauma, medicine, surgery, medical-surgical) in a university hospital in the US	Quasi-experimental, before-and-after study. Data included: Patient Views on Nursing Care (232 patients, 70 family members pre-implementation; 178 patients, 72 family members post-implementation); Nursing Assessment	– Significantly higher scores were obtained after implementation on two items: "I made sure I knew who my nurse was" and "include in the NBH discussion." and "communicated important information about care from shift to Shift<br>– After implementation, 102 patients (42%) made positive comments about the NBH, whereas 24 (10%) indicated that the nursing staff did not use NBH, used it inconsistently, or used it only to introduce the oncoming nurse, which was	Moderate

SN	Author(s) and year	Aim(s)	Study context	Study design and participants	Results	Methodological quality
				of Shift Report (148 nurses pre, 98 post). Event databases used for falls and medication errors	explained by inconsistencies noted in the process of implementing the change<br>– After implementation, some nurses made positive comments such as "improves accountability" and "increases patient involvement." Others made negative comments. "too time consuming," "used inconsistently or not as designed," and "repetitive"<br>– Falls decreased from 20 to 13 per month (35% reduction); medication errors decreased from 20 to 10 (50% reduction)	
47.	Johnson, and Cowin [102]	To explore nurses' perspectives on NBH introduction and the use of written handover sheets	Medical and surgical wards at three metropolitan hospitals in Sydney, Australia.	Qualitative study using six focus groups with 30 intentionally selected nurses	– Some nurses actively involved patients in the NBH and found this appropriate, while others preferred handing over outside patient rooms.<br>– Confidential information was managed flexibly, with nurses often seeking patient or family permission.<br>– The introduction of a written handover sheet supported continuity of care within the team	Strong
48.	Mc-Murray et al. [103]	To examine patients' perspectives on participation in shift-to-shift	Two medical units in a hospital in Queensland, Australia	Qualitative study with a descriptive case study approach, which included interviews to 10	– Patients perceived that NBH acknowledged their right to be informed and personalized care, especially when introduced to new nurses<br>– Patients stated that the NBH was an	Strong

SN	Author(s) and year	Aim(s)	Study context	Study design and participants	Results	Methodological quality
		bedside nursing handover		patients who experienced NBH	opportunity to understand the team's expectations regarding their progress and care<br>– Some patients preferred passive participation, while nurses often encouraged active involvement, including clarifying expectations and giving opinions	
49.	Mc-Murray et al. [105]	To identify factors influencing change in two hospitals that moved from taped and verbal nursing handover to bedside handover	The study was conducted in six wards of 2 regional acute care hospitals in two different states of Australia, that developed um standard operating protocol for communication at NBH	Qualitative study with an ethnographic approach that included 532 semi-structured observations e 34 in-depth interviews conducted with a purposive sample of nursing staff involved in the handovers	– Being part of a quality improvement strategy seemed to influence staff members' attitudes and understanding<br>– Standardization of structures and processes was a highly valued outcome for the nurses from both hospitals.<br>– Some difficulties during transitions were related to personalities and/or preferences.	Strong
50.	Greaves [117]	To explore how patients perceive the practice of nurses handing	A hospital within the National Health Service in England	Qualitative study with semi-structured interviews of 4 patients	– Patients reported that handovers took place outside their rooms and expressed a desire for more involvement to access information about their condition. Despite this, most preferred a passive	Moderate

SN	Author(s) and year	Aim(s)	Study context	Study design and participants	Results	Methodological quality
		over care at their bedside.			rather than active role.<br>– Most patients did not express concerns about information confidentiality.	
